# Computerized decision support to optimally funnel patients through the diagnostic pathway for dementia

**DOI:** 10.1186/s13195-024-01614-5

**Published:** 2024-11-26

**Authors:** Aniek M. van Gils, Antti Tolonen, Argonde C. van Harten, Sinthujah Vigneswaran, Frederik Barkhof, Leonie N. C. Visser, Juha Koikkalainen, Sanna-Kaisa Herukka, Steen Gregers Hasselbalch, Patrizia Mecocci, Anne M. Remes, Hilkka Soininen, Afina W. Lemstra, Charlotte E. Teunissen, Linus Jönsson, Jyrki Lötjönen, Wiesje M. van der Flier, Hanneke F. M. Rhodius-Meester

**Affiliations:** 1 Alzheimer Center Amsterdam and Department of Neurology, VU University Medical Center, Amsterdam UMC, De Boelelaan 1118, Amsterdam, 1081 HZ The Netherlands; 2 Amsterdam Neuroscience, Neurodegeneration, Amsterdam, 1081HV The Netherlands; 3 Combinostics Ltd, Tampere, Finland; 4 Department of Radiology & Nuclear Medicine, Vrije Universiteit Amsterdam, Amsterdam UMC, Amsterdam, 1081HV The Netherlands; 5 Queen Square Institute of Neurology and Centre for Medical Image Computing, University College London, London, UK; 6 Department of Medical Psychology, Amsterdam UMC, Amsterdam, 1081HV The Netherlands; 7 Amsterdam Public Health, Quality of Care, Amsterdam, 1081HV The Netherlands; 8 Division of Clinical Geriatrics, Center for Alzheimer Research, Department of Neurobiology, Care Sciences and Society, Karolinska Institutet, Stockholm, Sweden; 9 Institute of Clinical Medicine/Neurology, University of Eastern Finland, Kuopio, Finland; 10 Danish Dementia Research Centre, University of Copenhagen, Blegdamsvej 9, 2100 RigshospitaletCopenhagen, Denmark; 11 Division of Gerontology and Geriatrics, Department of Medicine and Surgery, University of Perugia, Piazzale Gambuli 1, 06129 Perugia, Italy; 12 Division of Clinical Geriatrics, Department of Neurobiology, Care Sciences and Society, Karolinska Institutet, Stockholm, SE Sweden; 13 Research Unit of Clinical Medicine, Neurology, University of Oulu, 90014 Oulu, Finland; 14 Department of Clinical Chemistry, Neurochemistry Laboratory, Amsterdam Neuroscience, Vrije Universiteit Amsterdam, Amsterdam UMC, Amsterdam, 1081HV The Netherlands; 15 Division of Neurogeriatrics, Department of Neurobiology, Care Sciences and Society, Karolinska Institutet, Stockholm, Sweden; 16 Department of Epidemiology and Data Sciences, Vrije Universiteit Amsterdam, Amsterdam UMC, Amsterdam, 1081HV the Netherlands; 17 Department of Internal Medicine, Geriatric Medicine Section, Amsterdam Cardiovascular Sciences Institute, Vrije Universiteit Amsterdam, Amsterdam UMC, Amsterdam, 1081HV The Netherlands; 18 Department of Geriatric Medicine, The Memory Clinic, Oslo University Hospital, 0379 Oslo, Norway

**Keywords:** Data-driven diagnosis, Differential diagnosis, Alzheimer’s disease, Eligibility

## Abstract

**Background:**

The increasing prevalence of dementia and the introduction of disease-modifying therapies (DMTs) highlight the need for efficient diagnostic pathways in memory clinics. We present a data-driven approach to efficiently guide stepwise diagnostic testing for three clinical scenarios: 1) syndrome diagnosis, 2) etiological diagnosis, and 3) eligibility for DMT.

**Methods:**

We used data from two memory clinic cohorts (ADC, PredictND), including 504 patients with dementia (302 Alzheimer’s disease, 107 frontotemporal dementia, 35 vascular dementia, 60 dementia with Lewy bodies), 191 patients with mild cognitive impairment, and 188 cognitively normal controls (CN). Tests included digital cognitive screening (cCOG), neuropsychological and functional assessment (NP), MRI with automated quantification, and CSF biomarkers. Sequential testing followed a predetermined order, guided by diagnostic certainty. Diagnostic certainty was determined using a clinical decision support system (CDSS) that generates a disease state index (DSI, 0–1), indicating the probability of the syndrome diagnosis or underlying etiology. Diagnosis was confirmed if the DSI exceeded a predefined threshold based on sensitivity/specificity cutoffs relevant to each clinical scenario. Diagnostic accuracy and the need for additional testing were assessed at each step.

**Results:**

Using cCOG as a prescreener for 1) syndrome diagnosis has the potential to accurately reduce the need for extensive NP (42%), resulting in syndrome diagnosis in all patients, with a diagnostic accuracy of 0.71, which was comparable to using NP alone. For 2) etiological diagnosis, stepwise testing resulted in an etiological diagnosis in 80% of patients with a diagnostic accuracy of 0.77, with MRI needed in 77%, and CSF in 37%. When 3) determining DMT eligibility, stepwise testing (100% cCOG, 83% NP, 75% MRI) selected 60% of the patients for confirmatory CSF testing and eventually identified 90% of the potentially eligible patients with AD dementia.

**Conclusions:**

Different diagnostic pathways are accurate and efficient depending on the setting. As such, a data-driven tool holds promise for assisting clinicians in selecting tests of added value across different clinical contexts. This becomes especially important with DMT availability, where the need for more efficient diagnostic pathways is crucial to maintain the accessibility and affordability of dementia diagnoses.

**Supplementary Information:**

The online version contains supplementary material available at 10.1186/s13195-024-01614-5.

## Introduction

Due to the increasing prevalence of dementia and disease-modifying therapies (DMTs) becoming available for patients, there is a high demand for accurate diagnosis, whilst keeping the diagnostic work-up efficient [1]. Accurate diagnosis is important to organize appropriate care and aid in future planning [2, 3]. With the availability of DMTs, an accurate etiological diagnosis is a prerequisite for deciding whether or not to initiate treatment [4].

Timely diagnosis and early identification of patients eligible for treatment require biomarkers of amyloid pathology [5, 6]. Currently available biomarkers for Alzheimer’s disease (AD) pathology (i.e., CSF, PET) are invasive and expensive, and healthcare systems are not ready for the anticipated changes due to a lack of resources and available expertise [7–9]. In regular clinical settings, only a small fraction of potentially eligible patients receive a biomarker-based confirmatory diagnosis [10]. Other frequently used tests for dementia diagnosis include cognitive screening, neuropsychological test batteries and magnetic resonance imaging (MRI) [11].

Despite the ongoing transition to a biological rather than a clinical disease model, clinicians in memory clinics may prioritize adequate syndrome diagnosis for appropriate care organization in certain patient populations. Therefore, clinical contexts, such as a focus on syndrome diagnosis or etiological diagnosis, may vary among clinicians due to the nature of their patient population and are also influenced by patients’ preferences and needs [12]. The added value of a specific diagnostic test depends on the clinical context. For example, while CSF sampling may not be necessary for syndrome diagnosis, it is often necessary for determining etiology and crucial for establishing a patient's eligibility for DMT [5, 13]. The increasing demand for accurate diagnosis, along with an anticipated shortage of trained dementia specialists, requires alternative approaches to keep dementia diagnosis accessible and affordable [8, 14].

To reduce the workload of both primary care physicians and dementia specialists, digital cognitive tests that can be administered to patients at home are promising [15]. Another solution is the use of a computerized decision support system (CDSS), an application that analyzes and visualizes all data to help healthcare providers make decisions and enhance patient care [16]. Our previous research demonstrated that a CDSS in memory clinics increased clinicians' confidence in diagnosis [17]. Furthermore, a CDSS can aid clinicians in making diagnostic decisions and funnel patients through the optimal diagnostic pathway [7, 13]. Therefore, a CDSS could be a cost-effective solution to assist clinicians in choosing tests relevant to the clinical scenario at hand, without compromising diagnostic accuracy [18].

A European task force recently defined a patient-centered biomarker-based workflow for specialized outpatient services, to aid clinicians in selecting the right biomarker at the right time [19]. Although this workflow is a positive development, clinicians still need to decide which diagnostic tool to use and when to use it. We add to such an expert-based workflow by presenting a data-driven approach to stepwise diagnostic testing, with the objective of assessing whether the use of such an approach holds promise for effectively and efficiently guiding diagnostic decision-making for three common clinical scenarios: syndrome diagnosis, etiological diagnosis, and potential DMT eligibility.

## Methods

### Study design and clinical scenarios

**Fig. 1:**
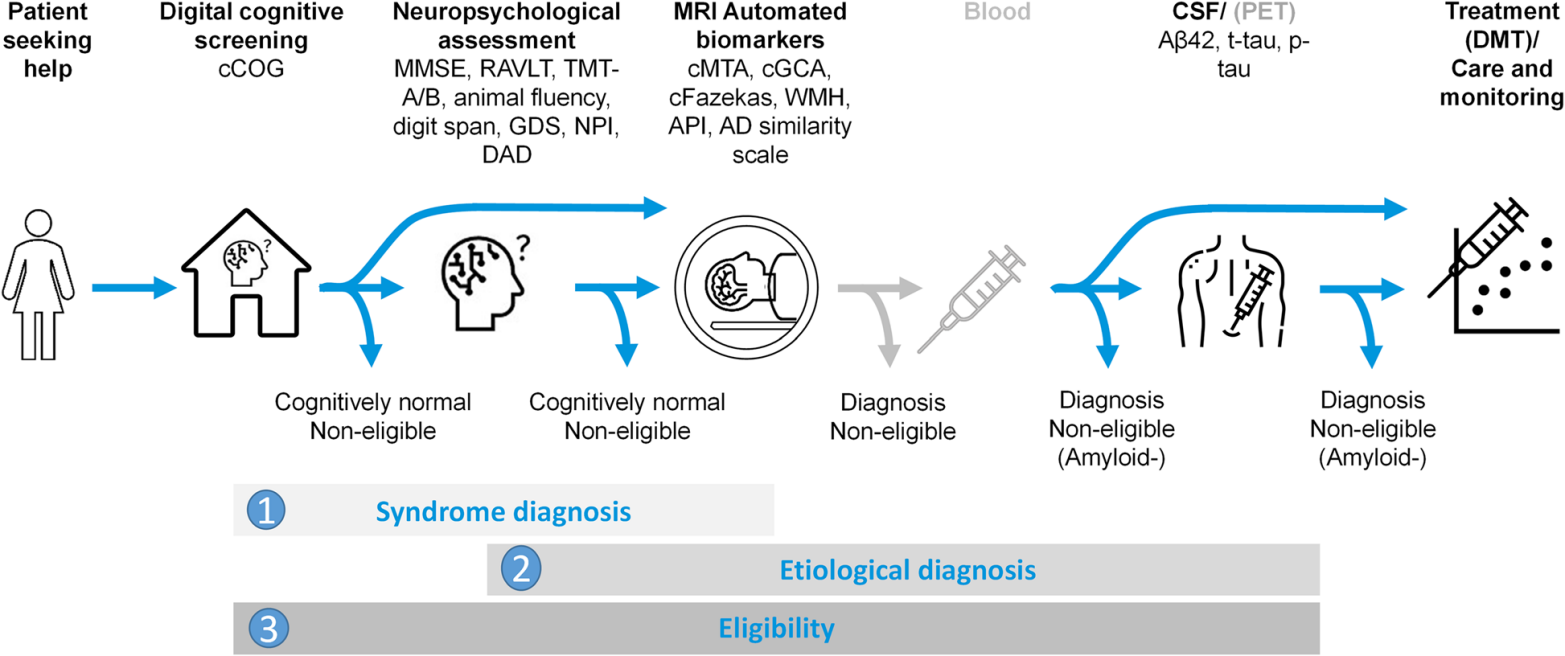
Clinical scenarios and the respective diagnostic tests used for each scenario. Note: blood/PET in light grey mean that these tests could be considered (in the future) in these steps, but they are not included in the current study. 1) Syndrome diagnosis, considering a diagnosis of CN, MCI or dementia. 2) Etiological diagnosis, considering a diagnosis of AD dementia, FTD, VaD, or DLB. 3) DMT eligibility, considering whether a patient would be eligible for DMT, based on appropriate use criteria by Cummings [6]. *Abbreviations*: cCOG: computerized cognitive test, MMSE: Mini-mental state examination, RAVLT: Rey-auditory verbal learning task, TMT-A/B: trail making test A/B, GDS: geriatric depression scale, DAD: disability assessment for dementia, MRI: magnetic resonance imaging, cMTA: computerized medial temporal lobe atrophy, cGCA: computerized global cortical atrophy, WMH: white matter hyperintensities, APS: anterior–posterior score, CSF: cerebrospinal fluid, Aβ42: amyloid β1-42, t-tau: total tau, p-tau: phosphorylated-tau, DMT: disease-modifying therapy

Figure 1 illustrates the operationalization of the three clinical scenarios and their respective diagnostic tests. Note that blood/PET in light gray means that these tests may be considered (in the future) in the diagnostic trajectory but are not included in the current study. A data-driven CDSS [17] was used to determine the probability of the diagnosis by generating a disease state index probability score (DSI, 0–1) through the weighted combination of diagnostic test results [20], including digital cognitive screening, neuropsychological and functional assessment, MRI, and CSF biomarkers. A subsequent test is only performed if the diagnosis is inconclusive based on the probability for diagnosis in the previous step.

The first scenario involves syndrome diagnosis, including a diagnosis of CN, MCI, or dementia, which is often used in a primary care or (local) memory clinic setting, where the focus is mostly on arranging care. Diagnostic tests included the (digital) cognitive screening test, cCOG [15], followed by neuropsychological assessment (NP), and MRI. For the second scenario, etiological diagnosis, encompassing differential diagnosis of AD, FTD, VaD, and DLB, diagnostic tests included cognitive assessment (cCOG, NP), MRI, and CSF analysis. In the third scenario, assessing potential eligibility for DMT according to the appropriate use criteria by Cummings [6], sequential diagnostic tests, including cCOG, NP, and MRI, were used to detect patients who should undergo CSF confirmation. Below, each diagnostic test battery is described in detail.

### Study participants

A total of 883 study participants from two memory clinic cohorts were included. From the Amsterdam Dementia Cohort (ADC) [21], we included data from 758 participants collected between 2004 and 2022, and from the PredictND cohort [17], we included data from 125 participants collected between 2015 and 2016 in three European memory clinics. All participants received a standardized multidisciplinary diagnostic work-up, including medical history, physical and neurological examination, cognitive and functional assessment, laboratory tests, brain imaging and CSF measurements. Participants were included if both brain MRI and CSF results were available. In the PredictND cohort, patients received a 12-month follow-up to confirm the diagnosis.

We included patients who were diagnosed with dementia (*n* = 504) due to Alzheimer’s disease (AD, *n* = 302), frontotemporal dementia (FTD, *n* = 107), vascular dementia (VaD, *n* = 35), or dementia with Lewy bodies (DLB, *n* = 60), representing the most prevalent patient groups in clinical practice. Diagnoses were made according to the criteria for probable AD [22, 23], FTD [24], VaD [25], and DLB [26]. Additionally, we included individuals with mild cognitive impairment (MCI, *n* = 191) [27]. Individuals who did not meet the criteria for MCI or dementia were diagnosed with SCD [28] and served as the cognitively normal (CN) group (*n* = 188). All clinical diagnoses were made in tertiary memory clinics through a consensus meeting in the ADC and after a 12-month follow-up in the PredictND cohort. Table 1 presents the clinical characteristics of the patients included in this study. All patients provided written informed consent for their data to be used for research purposes.

**Table 1:** Demographic and clinical characteristics for the pooled ADC and PredictND cohort (*n* = 883) according to diagnosis

	**N**	**CN *n* = 188**	**MCI *n* = 191**	**ADD *n* = 302**	**FTD *n* = 107**	**VaD *n* = 35**	**DLB *n* = 60**
**Demographics**							
Age, years	883	61 ± 8	66 ± 8	67 ± 78	63 ± 7	70 ± 8	69 ± 8
Sex, female	883	80 (42.6)	70 (36.6)	163 (54.0)	46 (43.0)	11 (31.4)	8 (13.3)
Education, years	836	13 [10-17]	12 [9-14]	10 [9-13]	10 [9-13]	10 [9-13]	10 [9-13]
**Digital cognitive screening, cCOG**							
cCOG, learning	111	23 ± 8	19 ± 4	15 ± 5	17 ± 6	19 ± NA	15 ± 8
cCOG, recall	110	9 ± 2	6 ± 2	4 ± 2	7 ± 5	8 ± NA	6 ± 3
cCOG, TMT-a (s)	110	48 ± 16	65 ± 22	77 ± 32	163 ± 173	63 ± NA	84 ± 35
cCOG, TMT-b (s)	104	171 ± 98	231 ± 82	322 ± 153	153 ± NA	283 ± NA	246 ± 91
**Neuropsychology**							
MMSE [0–30]	879	29 [28-30]	27 [25-29]	23 [19-25]	25 [23-27]	26 [23-27]	24 [22-26]
Memory, learning	839	42 ± 9	31 ± 8	22 ± 8	28 ± 8	24 ± 8	25 ± 9
Memory, recall	839	9 ± 3	4 ± 3	2 ± 2	4 ± 3	3 ± 3	4 ± 3
TMT-A (s)	861	36 ± 14	48 ± 19	72 ± 39	59 ± 30	90 ± 37	92 ± 38
TMT-B (s)	710	85 ± 36	133 ± 67	191 ± 82	161 ± 77	224 ± 82	218 ± 87
Animal fluency	855	23 ± 6	19 ± 5	13 ± 6	12 ± 7	11 ± 5	13 ± 6
**Neuropsychiatric symptoms**							
GDS [0–15]	783	3 ± 3	3 ± 3	3 ± 2.3	3 ± 3	4 ± 3	4 ± 3
NPI, total score	706	8 ± 10	10 ± 11	11 ± 10	21 ± 17	14 ± 9	14 ± 12
**Functional assessment**							
DAD [0–100]	510	100 [92–100]	93 [86–98]	88 [75–96]	83 [63–97]	70 [66–83]	77 [60–96]
**Automated MRI biomarkers**							
cMTA, left	883	0.35 ± 0.61	0.94 ± 1.00	1.53 ± 0.92	2.07 ± 1.33	1.79 ± 1.24	1.08 ± 0.75
cMTA, right	883	0.37 ± 0.58	0.96 ± 0.99	1.48 ± 0.93	1.72 ± 1.27	1.73 ± 1.10	1.07 ± 0.85
cGCA	882	0.51 ± 0.58	0.80 ± 0.71	1.30 ± 0.74	1.37 ± 0.68	1.70 ± 0.68	1.19 ± 0.59
cFazekas	881	0.65 ± 0.66	1.15 ± 0.93	1.06 ± 0.80	0.90 ± 0.81	2.64 ± 0.53	1.01 ± 0.75
WMH, volume	881	3.71 ± 6.23	10.58 ± 18.83	8.29 ± 11.85	6.36 ± 11.40	50.54 ± 31.58	6.08 ± 6.99
Anterior–posterior score	883	0.35 ± 1.21	0.01 ± 1.29	0.30 ± 1.68	-2.68 ± 2.39	0.28 ± 1.52	0.42 ± 1.37
AD similarity	882	0.39 ± 0.10	0.47 ± 0.12	0.60 ± 0.11	0.44 ± 0.12	0.60 ± 0.10	0.51 ± 0.10
**Cerebrospinal fluid**							
Aβ_42_, pg/ml	883	1034 ± 246	830 ± 297	617 ± 164	1017 ± 266	798 ± 215	845 ± 249
Total tau, pg/ml	883	284 ± 152	446 ± 274	717 ± 413	383 ± 205	302 ± 163	311 ± 158
p-tau, pg/ml	883	48 ± 18	65 ± 32	86 ± 38	49 ± 22	42 ± 20	47 ± 21

Data presented in mean ± SD, median [IQR], or n(%)

*Abbreviations*: *CN* cognitively normal, *MCI* mild cognitive impairment, *ADD* Alzheimer’s disease dementia, *FTD* frontotemporal dementia, *VaD* vascular dementia, *DLB* dementia with Lewy bodies, *MMSE* Mini-Mental state Examination, *cCOG* computerized cognitive test, *TMT* Trail Making Test, *GDS* geriatric depression scale, *NPI* neuropsychiatric inventory, *DAD* disability assessment for dementia, *cMTA* computed medial temporal lobe atrophy, *cGCA* computed global cortical atrophy, *WMH* white matter hyperintensities, *Aβ*_*42*_ β-amyloid 1–42, *p-tau* phosphorylated tau

### Digital cognitive screening

We aimed to apply a future-proof stepwise approach and thus incorporated a digital cognitive screening test, simulating a situation in which cognitive screening can start at home. As a part of the PredictND study, a subset of patients (*n* = 111, 13%) completed a digital cognitive screening test. cCOG is a web-based test tool that can accurately detect MCI and dementia [15]. cCOG has a completion time of 20 min and consists of a memory learning and recall task, and modified trail-making tests A and B. For the patients who had not performed cCOG, we simulated the results for each task from their neuropsychological equivalents.

### Neuropsychological assessment

Data from the following neuropsychological tests were included: the Mini-Mental State Examination (MMSE) was performed to assess global cognitive function [29]. The Rey auditory verbal learning task and the Consortium to Establish a Registry for Alzheimer’s Disease word list memory test were used to assess learning and recall [30, 31], the Trail Making Test A and B (TMT-A/B) for mental processing speed and executive function [32], animal fluency for language and executive function [33], and forward and backward digit span for attention and executive functioning [34]. To assess neuropsychiatric symptoms, we used the Geriatric Depression Scale (GDS) [35] and the Neuropsychiatric Inventory (NPI, [36]). Missing data ranged from 177 (20%) for the NPI to 4 (0.5%) for the MMSE. To assess functional decline, the disability assessment for dementia (DAD) [37] was used, for which we missed data on *n* = 373 (42%).

### MRI acquisition and automated biomarkers

MRI data were acquired using 1.5 or 3 T scanners. Three-dimensional T1-weighted gradient echo sequence and fast fluid-attenuated inversion recovery (FLAIR) sequence images were used. In this study, we used automated biomarkers obtained with the cMRI quantification tool as described in [38, 39]. The automated imaging biomarkers included: computed medial temporal lobe atrophy (cMTA): which was calculated from hippocampal volumes and inferior lateral ventricles from both hemispheres, and obtained using a multi-atlas segmentation algorithm [38, 40]. Computed global cortical atrophy (cGCA): grey matter concentration measured by voxel-based morphometry analysis [40]. The white matter hyperintensities (WMH) volume was automatically extracted from FLAIR images. The computed Fazekas (cFazekas) was estimated from these volumes combined with deep WMH [38, 40]. The anterior–posterior score (APS) is the ratio of cortical volumes in the frontal and temporal lobes relative to those in the parietal and occipital lobes, providing a specific measure for characterizing frontotemporal atrophy [39]. The AD similarity scale was derived by representing the region of interest (ROI) in the patient image as a linear combination of the corresponding ROIs from a database of previously diagnosed patients [13]. All imaging markers were corrected for head size, age, and sex.

### Fluid biomarkers

The CSF biomarkers amyloid β1-42 (Aβ42), total tau (t-tau), and phosphorylated tau (p-tau) were measured locally with commercially available enzyme-linked immunosorbent assays (ELISA) (Innotest®, Fujirebio and Elecsys, Roche). Elecsys results were mapped to Innotest according to [41]. A drift-corrected cutoff of < 813 pg/ml was applied to determine Aβ_42_ abnormalities [42], and > 375 pg/ml was applied for total tau abnormalities [43]. AD pathology was defined using the total-tau/Aβ_42_ ratio with a cutoff of ≥ 0.46 [44].

### Simulating stepwise testing for different clinical scenarios

#### Disease state index probability

To predict the diagnosis at each step, we used the disease state index (DSI) classifier. The DSI is a simple, data-driven, machine-learning method that compares different diagnostic (either syndrome or etiological) groups with each other (e.g., CN vs. dementia, or AD dementia vs. VaD) based on a training set of diagnosed patients. The DSI was previously validated in the European PredictND project [17]. For each diagnostic test, the patient data are compared to the distributions of the diagnostic groups in the training set, yielding a scalar index between zero and one that indicates the probability of a specific diagnosis [45]. Patients with low or high DSI values are typically more likely to be correctly classified than patients with intermediate DSI values. The DSI handles different types of variables, such as demographic information, cognitive test results, CSF biomarkers, and MRI data, and tolerates missing data. To reflect real-world practice, we included patients with missing data on neuropsychological tests [20]. The dataset was normalized according to age and sex. Tenfold cross-validation was performed with ten different test/train set divisions. Each time, 10% of the individuals were used as the test set and the remaining 90% were used as the training set. The test sets were separated, meaning that each subject appeared in exactly one test set and nine training sets in each round of cross-validation. The results over the ten cross-validations were combined and averaged to obtain the final result. The method is described in detail in the supplementary files Appendix A.

#### Probability cutoffs

In this study, the probability cutoffs varied depending on the clinical scenario. Cutoffs were determined visually by plotting sensitivity and specificity against probability cutoff values for each step in each scenario (see supplementary files: Supplementary Figs. 1–5). In clinical practice, there are established cutoffs for certain tests, such as amyloid positivity. However, decisions based on combined data from multiple sources and clinical impressions rely on the confidence of the clinician. Clinicians make decisions based on their confidence level and they may request additional testing or delay the diagnosis if they lack confidence. Confidence is subjective and depends on factors such as the clarity of the findings, the data available, the clinicians’ expertise, and their personality. Probability cutoffs aim to make the decision process more objective when interpreting all acquired data.

It is important to note that the CDSS does not consider clinical impressions, which are an essential part of the diagnostic process, so it should be considered supportive, as diagnosis is ultimately a clinical judgment. Additionally, there is a trade-off between accuracy and the number of tests. Acquiring more data can increase confidence and accuracy, but it also comes at a cost. Finally, decisions are always a compromise between sensitivity and specificity, i.e., how we value false positives and false negatives. For syndrome diagnosis, high sensitivity was considered important for minimizing the number of false-negative cases, while a balance between sensitivity and specificity was chosen for accurate etiological diagnosis.

#### Clinical scenarios

Figure 1 shows the diagnostic tests considered for each diagnostic scenario. Diagnostic strategies followed a predetermined order based on clinical guidelines and current practice. At each step, DSI values were calculated for the combination of diagnostic tests used. At each step, we assessed whether the DSI values exceeded the predetermined cutoff. If DSI values exceeded the cutoff, a diagnosis was made, and the diagnostic process was stopped. For patients for whom the diagnosis remained uncertain, i.e., for whom the DSI did not exceed the cutoff, additional tests were added to the diagnostic trajectory.

We used letter value plots [46] to depict the distribution of DSI values among different diagnostic groups. Letter value plots provide statistical insights in a visually intuitive manner. Each group is visually represented by boxes. The length of the box represents the interquartile range (IQR), which contains the middle 50% of the data in the group.

##### Scenario 1: syndrome diagnosis

For scenario 1, syndrome diagnosis, we included all patients. We used a pairwise classifier (‘CN’ vs. ‘dementia’). A DSI close to one increases the likelihood of a dementia diagnosis, and a DSI close to zero suggests CN. Patients with intermediate DSI values were considered to have MCI in the final step. We divided the scenario into two different pathways:

- Scenario 1A: Cognitive and functional assessment (NP)—The approach used in clinical practice today (base case). In choosing the cutoffs, we aimed to reflect decision making in clinical practice and used a DSI cutoff of 0.3 for CN and 0.7 for dementia patients. Patients with a DSI > 0.3 would require additional MRI testing.
  - Step 1: NP
    - ▪ DSI (CN/dem) < 0.3: CN; no next test
    - ▪ DSI (CN/dem) 0.3–0.7: MCI; next test, MRI
    - ▪ DSI (CN/dem) > 0.7: dementia; next test, MRI

- Scenario 1B: To enhance the current base case, in scenario 1B we used the digital cCOG as a prescreening step. We aimed for detecting clearly cognitively normal participants (DSI < 0.1, low probability) and patients with clear cognitive problems (DSI > 0.95, high probability), while the patients in between require additional NP testing (intermediate probability). For steps 2 and 3, the cutoff values were 0.3 for CN and 0.7 for dementia patients.
  - Step 1: cCOG
    - ▪ DSI (CN/dem) < 0.1: CN; no next test
    - ▪ DSI (CN/dem) 0.1–0.95: indeterminate result, next test, NP
    - ▪ DSI (CN/dem) > 0.95: dementia, next test, MRI
  - Step 2: cCOG + NP
    - ▪ DSI (CN/dem) < 0.3: CN; no next test
    - ▪ DSI (CN/dem) 0.3–0.7: MCI; next test, MRI
    - ▪ DSI (CN/dem) > 0.7: dementia; next test, MRI

##### Scenario 2: etiological diagnosis

For the purpose of etiological diagnosis, we excluded patients with MCI because the question of differential diagnosis only becomes relevant at the stage of dementia. We included 1) cognitive testing using cCOG and NP, 2) MRI, and 3) CSF. For step 1, we used the two-class DSI classifier (‘CN’ vs. ‘dementia’) with a low cutoff (DSI < 0.25) to prioritize sensitivity. Only after MRI can differential diagnosis be performed. Here, we used a multiclass DSI classifier, averaging the DSI value of each of the etiological groups against all other groups (i.e., for AD it is the average of: FTD vs. AD, DLB vs. AD, and VaD vs. AD). In this way, a DSI value (continuous value between zero and one) is provided for each patient and each diagnostic group (AD-FTD-VaD-DLB), estimating the probability of the specific diagnosis. The highest average DSI value defines the most likely class for the patient. For steps 2 and 3, we set the DSI cutoff at > 0.6 to balance between the number of patients who could be given a diagnosis and the accuracy of that diagnosis. As soon as the DSI ≥ 0.6 for any etiological diagnosis was reached the diagnostic process was concluded. If the DSI remained < 0.6, the data did not support a confident diagnosis. Additional data, such as CSF in step 3 or other tests, such as more detailed neuropsychological testing, electroencephalography (EEG), FDG-PET, or DaT-scan are required (not addressed in this paper).

- Step 1: cCOG & NP
  - ▪ DSI (CN/dem) ≤ 0.25: CN, no next test
  - ▪ DSI (CN/dem) > 0.25: dementia; next test, MRI
- Step 2: cCOG & NP + MRI
  - ▪ DSI (multiclass) > 0.6 for AD, FTD, VaD, DLB: diagnosis; no next test
  - ▪ DSI (multiclass) ≤ 0.6 for AD, FTD, VaD, DLB: next test: CSF
- Step 3: cCOG & NP + MRI + CSF
  - ▪ DSI (multiclass) > 0.6 for AD, FTD, VaD, DLB: diagnosis; no next test
  - ▪ DSI (multiclass) ≤ 0.6 for AD, FTD, VaD, DLB: next test or follow-up required (not addressed)

##### Scenario 3: DMT eligibility

In scenario 3, we included all patients and defined potential DMT eligibility according to Cummings' appropriate use recommendations [6] and the available data in the dataset. Potential eligibility was defined as a diagnosis of MCI/dementia due to AD, with MMSE ≥ 22, cFazekas < 2.5, and positive amyloid biomarkers. According to these criteria, our dataset consisted of a total of 230 potentially eligible patients. The goal of this scenario is to perform a minimum number of CSF tests while identifying a maximum number of eligible patients. We applied the following steps to select patients for confirmatory CSF testing: 1) cCOG, 2) NP, and 3) MRI. Unlike scenarios 1 and 2, the gold standard for diagnosis in this case is amyloid status based on CSF rather than clinical diagnosis. Step 4 involves CSF testing for confirmation of the biomarkers status. For steps 1 and 2, we used a pairwise classifier (CN vs. dementia). At step 1, the DSI threshold was set at 0.1 All patients with a DSI > 0.1 proceeded to step 2, involving NP. Here, the threshold increased to 0.3. All patients who were predicted to be ‘potentially eligible’ after the first two steps continued to step 3, MRI. For step 3, we used a different pairwise classifier (‘AD’, ‘other’), with a cutoff of 0.1, to select patients for CSF. A cutoff of < 0.1 points for a non-AD diagnosis and amyloid confirmation is not needed.

- Step 1: cCOG
  - ▪ DSI (CN/dem) ≤ 0.1: CN, not eligible, no next test
  - ▪ DSI (CN/dem) > 0.1: probable eligible; next test, NP
- Step 2: cCOG + NP
  - ▪ DSI (CN/dem) ≤ 0.3: CN, not eligible, no next test
  - ▪ DSI (CN/dem) > 0.3: probable eligible; next test, MRI
- Step 3: cCOG + NP + MRI
  - ▪ If DSI (AD/other) < 0.1 and DSI for any of the other etiologies > 0.85: non-AD, not eligible, no next test
  - ▪ DSI (AD/other) > 0.1: probable AD and DSI for any of the other etiologies < 0.8, probably eligible; next test, CSF
- (Step 4: Confirmatory CSF testing)

### Statistical analyses

All statistical analyses were performed using R version 4.0.3. DSI analyses were performed using a Python implementation of DSI algorithm in Python 3.10.13.

The predicted diagnoses in scenarios 1 and 2 were compared to the clinical diagnoses as made in the respective memory clinics. In scenario 3, the predicted eligibility was compared to actual CSF results. For each scenario, we assessed the share of correct diagnoses (estimated by summing the number of true positive and true negative cases), sensitivity, specificity, and the need for additional testing at each step. The calculations for scenarios 1 and 3 were repeated using the subset of patients with real cCOG data.

Sub analyses were performed to compare the groups of patients with and without diagnosis using analysis of variance (ANOVA) and chi‐squared tests to evaluate differences between the groups in diagnosis, demographics, or clinical characteristics.

## Results

### Syndrome diagnosis

**Fig. 2:**
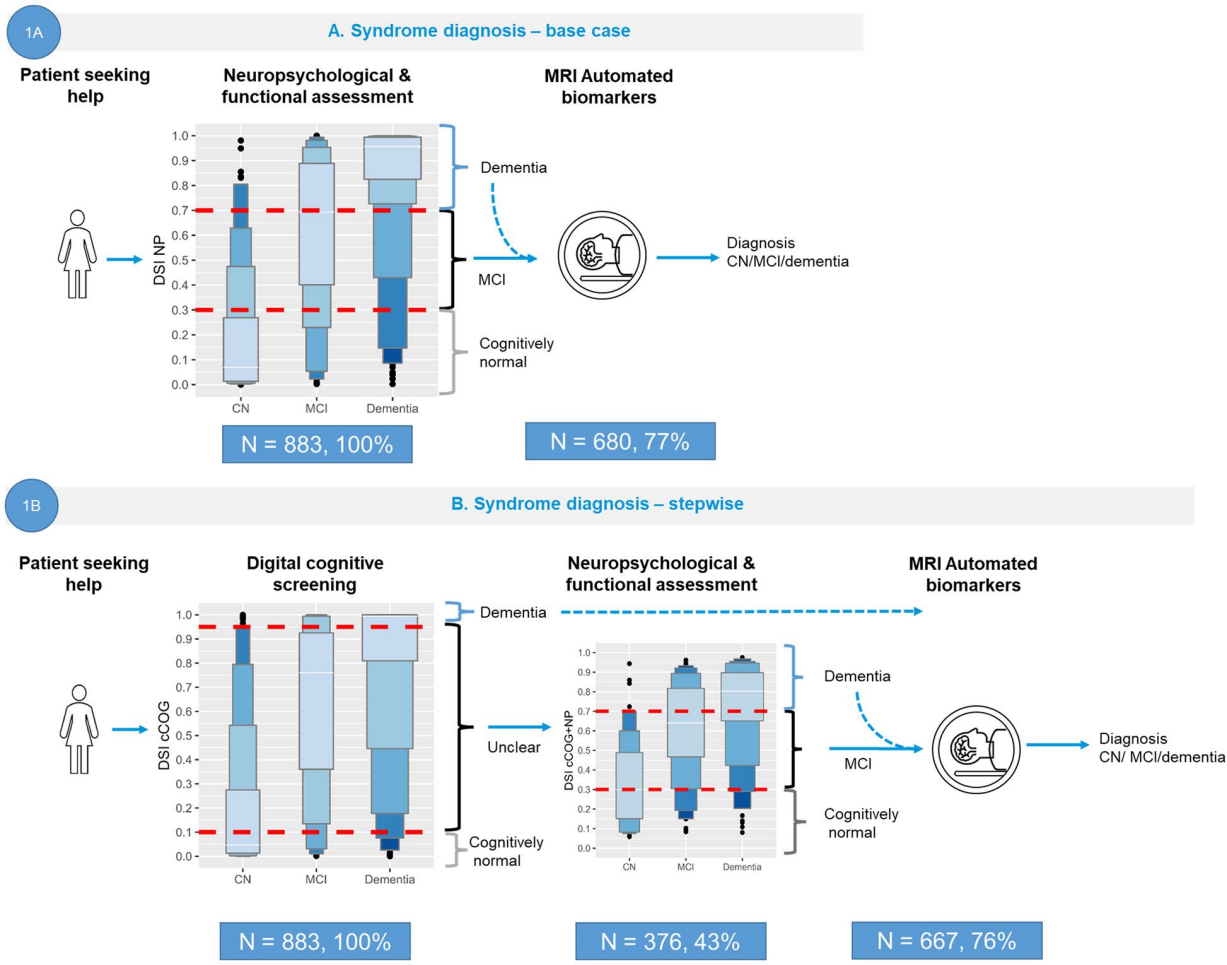
Visual representation of scenario 1, syndrome diagnosis, for scenario 1A) syndrome diagnosis using NP, and scenario 1B) stepwise syndrome diagnosis, making use of letter-value plots. NOTE: The figure includes letter-value plots for DSI scores. The length of the first box represents the interquartile range (IQR), which contains the middle 50% of the data in the group, the second box (E) 12.5% at each end, the third box covers (D) 6.25% on each end, and the fourth box (C) 3.13%. The longer the box, the greater the variability. The percentages indicate the proportion of the total population that requires additional testing. Red dashed lines depict the DSI cutoff-vales. MCI: mild cognitive impairment, MRI: magnetic resonance imaging

Scenario 1 was divided into two sub scenarios that included all patients (N = 883). Scenario 1A represents the base case, where the syndrome diagnosis is based on NP. In scenario 1B, the digital cognitive test cCOG was added as a prescreener. A visual representation of both scenarios is shown in Fig. 2.

#### Scenario 1A. syndrome diagnosis using NP

When applying the cutoff of < 0.3 for CN and > 0.7 for dementia, 203 participants were classified as CN, 131 as MCI, and *n* = 549 as having dementia. Using this scenario led to a diagnostic accuracy of 0.74 and would require MRI in 77% of patients. Table 2 shows the corresponding confusion matrix.

**Table 2:** Confusion matrices for syndrome diagnosis scenarios 1A and 1B

	Scenario 1A. NP (all)					
	**CN**	**MCI**	**DEM**			
**CN**	147	32	24			
**MCI**	31	65	35			
**DEM**	10	94	445			
*Cutoffs*	< 0.3	0.3–0.7	> 0.7			
*Sensitivity*	0.92	0.34	0.88			
*Specificity*	0.78	0.90	0.73			
*Accuracy*	0.74					
	Scenario 1B. cCOG			Scenario 1B. cCOG + NP		
	**CN**	**MCI**	**DEM**	**CN**	**MCI**	**DEM**
**CN**	113	19	20	148	35	33
**MCI**	69	132	175	29	56	44
**DEM**	6	40	309	11	100	427
*Cutoffs*	< 0.1	0.1–0.95	> 0.95	< 0.3	0.3–0.7	> 0.7
*Sensitivity*	0.94	0.69	0.61	0.90	0.29	0.85
*Specificity*	0.60	0.65	0.88	0.79	0.89	0.71
*Accuracy*		0.63			0.72	

In the confusion matrix each column represents the actual diagnosis and each row the diagnosis suggested by the classifier; the cells show the number of patients in each category

*Abbreviations*: *CN* cognitively normal, *MCI* mild cognitive impairment, *cCOG* computerized cognitive testing, *NP* neuropsychological and functional assessment, *MRI* magnetic resonance imaging

#### Scenario 1B. cCOG + NP + MRI

In scenario 1B, diagnostic tests were added sequentially to reduce NP testing to only when digital screening was insufficient and expanded with MRI when necessary. Table 2 shows the confusion matrices for the consecutive steps. In the first step, cognitive screening with cCOG, the threshold was low (< 0.1) for CN and high (> 0.95) for dementia. In this step, 152 participants were predicted to be CNs, and 355 were predicted to have dementia. Additional NP testing was added for the 376 participants with indeterminate DSI values (43%). The combination of the two steps resulted in a diagnostic accuracy of 0.71 (Table 3) and required MRI in 76% (*n* = 667). Supplementary Table 1 shows the results for the subset of patients for whom real cCOG data were available.

**Table 3:** Confusion matrix for patients receiving diagnosis after stepwise application of cCOG, cognitive testing, MRI and CSF

	**CN**	**AD**	**FTD**	**VaD**	**DLB**
**CN**	141	4	12	1	3
**AD**	4	182	3	2	3
**FTD**	14	23	51	2	2
**VaD**	2	12	4	19	1
**DLB**	5	23	8	2	33
*Cutoffs*	> 0.6	> 0.6	> 0.6	> 0.6	> 0.6
*Sensitivity*	0.85	0.75	0.65	0.73	0.79
*Specificity*	0.93	0.95	0.90	0.96	0.91
*Total accuracy*			0.77		

In the confusion matrix each column represents the actual diagnosis and each column the diagnosis suggested by the classifier; the cells show the number of patients in each category

*Abbreviations*: *CN* cognitively normal, *AD* Alzheimer’s disease, *FTD* Frontotemporal dementia, *VaD* vascular dementia, *DLB* dementia with Lewy bodies

### Etiological diagnosis

**Fig. 3:**
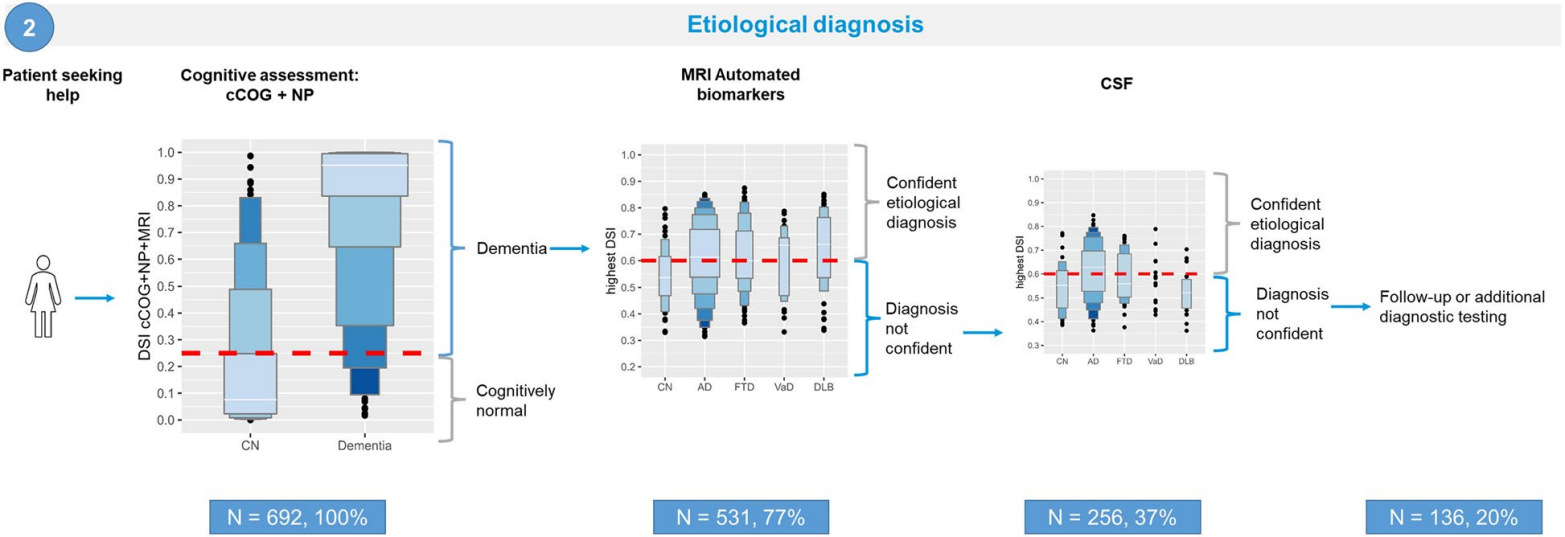
Visual representation of scenario 2, stepwise etiological syndrome diagnosis, making use of letter-value plots. NOTE: The figure includes letter-value plots for DSI scores. The length of the first box represents the interquartile range (IQR), which contains the middle 50% of the data in the group, the second box (E) 12.5% at each end, the third box covers (D) 6.25% on each end, and the fourth box (C) 3.13%. The longer the box, the greater the variability. The percentages indicate the proportion of the total population that requires additional testing. Red dashed lines depict the DSI cutoff-vales. MCI: mild cognitive impairment, MRI: magnetic resonance imaging

For the second scenario, we included CN (*n* = 188) and dementia patients (302 AD, 107 FTD, 35 VaD, 60 DLB). We started with cognitive testing to distinguish between controls and dementia patients. Subsequently, we sequentially added MRI and CSF to determine etiology (Fig. 3).

Based on initial cognitive and functional testing, 162 individuals were identified as CN and 530 were identified as having dementia, with a sensitivity of 97% and specificity of 75% (DSI cutoff < 0.25; supplementary Table 2). Subsequently, 77% of the participants proceeded to the next step, which involved MRI. Following MRI, DSIs were calculated for each of the diagnostic groups (AD, FTD, VaD, DLB) for each individual patient (Supplementary Fig. 6). A DSI > 0.6 for any of the etiological diagnosis confirmed that diagnosis. Of the 531 patients who underwent MRI, 275 patients (52%) received a diagnosis (Supplementary Table 3), and the remaining 256 patients proceeded to the final step of CSF examination. Of the 256 patients who underwent CSF examination (Supplementary Fig. 6), 120 (47%) were ultimately diagnosed with sufficient confidence (DSI > 0.6) (Supplementary Table 4). The combination of all steps led to diagnosis in a total of 556 participants (80%) with an accuracy of 0.77, as shown in Table 3A.

After CSF testing, the diagnosis remained uncertain for 20% of the patients, requiring additional diagnostic testing or follow-up. Of these patients, 16% were CN, and 84% had dementia. The demographic and clinical characteristics of these patients are shown in Table 4. Compared to patients who received a diagnosis, patients without a diagnosis after the stepwise approach were more likely to have a clinical diagnosis of FTD or DLB, were older and had worse neuropsychological and neuropsychiatric assessment outcomes. Furthermore, patients without a diagnosis had a greater vascular burden on MRI (cFazekas, *p* = 0.032), suggesting the presence of mixed etiologies in these patients.

**Table 4:** Demographic and clinical characteristics for patients receiving a diagnosis (*n* = 556) or no diagnosis (*n* = 136) after stepwise cognitive testing, MRI, and CSF

	**Without diagnosis after stepwise approach *n* = 136**	**With diagnosis after stepwise approach *n* = 556**	*p-value*
**True diagnosis, n (%)**			**0.003**
CN^a^	22 (16)	166 (30)	
AD	58 (43)	244 (44)	
FTD^a^	29 (21)	78 (14)	
VaD^a^	9 (7)	26 (5)	
DLB	18 (13)	42 (8)	
**Demographics**			
Age, years	66 ± 8	65 ± 9	**0.044**
Sex, female	66 (48.5)	242 (43.5)	0.339
Education, years	13 [10-16]	15 [12-17]	0.080
**Neuropsychology**			
MMSE [0–30]	25 [22-27]	25 [22-28]	0.078
Memory, learning	26 ± 8	30 ± 12	**0.002**
Memory, recall	4 ± 3	5 ± 4	**0.015**
TMT-A (s)	73 ± 40	60 ± 36	** < 0.001**
TMT-B (s)	178 ± 91	145 ± 83	** < 0.001**
Animal fluency	14 ± 6	16 ± 8	**0.029**
**Neuropsychiatric symptoms**			
GDS [0–15]	4 ± 3	3 ± 2	**0.001**
NPI, total score	15 ± 14	12 ± 12	**0.011**
**Functional assessment**			
DAD [0–100]	78.0 ± 22.8	84.3 ± 18.3	**0.006**
**Automated MRI biomarkers**			
cMTA, left	1.38 ± 1.12	1.24 ± 1.11	0.169
cMTA, right	1.26 ± 1.01	1.17 ± 1.07	0.372
cGCA	1.19 ± 0.77	1.09 ± 0.77	0.166
cFazekas	1.14 ± 0.89	0.97 ± 0.85	**0.032**
WMH, volume	10.47 ± 16.58	8.26 ± 15.22	0.137
Anterior–posterior index	-0.33 ± 2.12	-0.09 ± 1.96	0.195
AD similarity	0.53 ± 0.13	0.51 ± 0.15	**0.048**
**Cerebrospinal fluid**			
Aβ_42_, pg/ml	824 ± 269	821 ± 293	0.925
Total tau, pg/ml	439 ± 277	5056 ± 378	0.057
p-tau, pg/ml	59 ± 29	65 ± 36	0.065

Data presented in mean ± SD, median [IQR], or n(%). ^a^Post hoc pairwise comparisons indicate group differences after false discovery rate correction

*Abbreviations*: *CN* cognitively normal, *MCI* mild cognitive impairment, *AD* Alzheimer’s disease, *FTD* frontotemporal dementia, *VaD* vascular dementia, *DLB* dementia with Lewy bodies, *MMSE* Mini-Mental state Examination, *RAVLT* Rey Auditory Verbal Learning Test, *TMT* Trail Making Test, *GDS* geriatric depression scale, *NPI* neuropsychiatric inventory, *DAD* disability assessment for dementia, *cMTA* computed medial temporal lobe atrophy, *cGCA* computed global cortical atrophy, *API* anterior posterior index, *WMH* white matter hyperintensities, *Aβ*_*42*_ β-amyloid 1–42, *p-tau* phosphorylated tau

### DMT eligibility

**Fig. 4:**
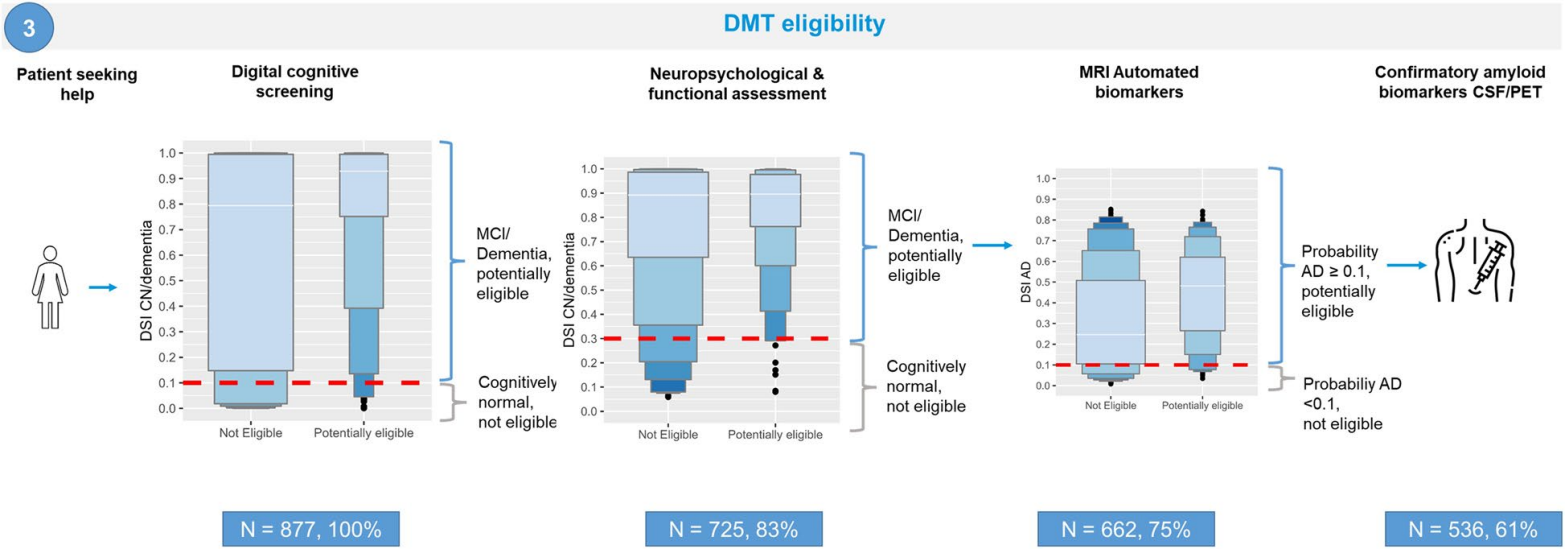
Visual representation scenario 3, stepwise eligibility testing, making use of letter-value plots. NOTE: The figure includes letter-value plots for DSI scores. The length of the first box represents the interquartile range (IQR), which contains the middle 50% of the data in the group, the second box (E) 12.5% at each end, the third box covers (D) 6.25% on each end, and the fourth box (C) 3.13%. The longer the box, the greater the variability. Red dashed lines depict the DSI cutoff-vales. *Abbreviations*: AD: Alzheimer’s disease, MCI: mild cognitive impairment, MRI: magnetic resonance imaging, CSF: cerebrospinal fluid, PET: positron emission tomography

Finally, we studied how a CDSS can efficiently support the determination of eligibility for DMT. We included patients with complete data on amyloid status, MMSE, and cFazekas score (*n* = 187 CN, *n* = 190 MCI, *n* = 500 dementia (all causes)). To determine potential eligibility for DMT, it is important to first detect the clinical stage, followed by the etiology. In contrast to scenarios 1 and 2, where clinical diagnosis was considered the gold standard, scenario 3 used amyloid status as determined in CSF as the gold standard. Therefore, CSF testing is not a separate step as in other scenarios, but rather is performed as a confirmatory test in all potentially eligible patients. The goal of this scenario is to perform a minimum number of CSF tests, while identifying a maximum number of eligible patients. Figure 4 shows a visual representation of the stepwise eligibility testing.

The first steps are used to identify MCI and dementia, since CN are not eligible for DMT. Based on digital cognitive screening, 152 patients were identified as non-eligible (DSI cutoff < 0.1). The remaining 725 patients (83%) continued to NP, after which 63 were identified as non-eligible (DSI cutoff < 0.3). Based on the cognitive assessment, 662 patients (75%) were selected for MRI as the next step to detect potential eligibility. After MRI, all patients with a DSI for AD > 0.1 and < 0.8 for any of the other etiologies (*n* = 528, 60%) were considered potentially eligible and were thus selected for confirmatory CSF testing. Of those patients, 191 met the eligibility criteria (MCI/dementia due to AD, MMSE ≥ 22, cFazekas < 2.5, and positive amyloid biomarkers) whereas 337 did not. Supplementary Table 5 shows the confusion matrix for each step for all patients (A) and the subset with real cCOG data (B), Table 5 shows the confusion matrix of all three steps combined. Stepwise diagnostic testing correctly selected 191/230 potentially eligible patients (true positive, 83%) for confirmatory CSF. Furthermore, 349 patients were identified as non-eligible, 310 of whom did not meet the eligibility criteria (true negative). The distribution of AD diagnoses of the 39 false negative patients is showed in Table 6, indicating that the sensitivity for detecting eligible patients with AD dementia is 90%. Half of the cases were stable MCI patients, who showed no disease progression in the 12-month follow up of the PredictND study.

**Table 5:** Confusion matrix for patients classified as potentially eligible after stepwise application of cCOG, cognitive testing and MRI

	**Not eligible**	**Potentially eligible**
**Not eligible**	310	39
**Potentially eligible**	337	191
*Sensitivity*	0.83	
*Specificity*	0.47	

In the confusion matrix each column represents the actual diagnosis and each row the diagnosis suggested by the classifier; the cells show the number of patients in each category. Note that potentially eligible patients are: AD or MCI with positive Aβ biomarkers, MMSE ≥ 22, and Fazekas < 2.5

**Table 6:** Distribution of AD diagnoses of patients classified as eligible in the stepwise approach

	**sMCI due to AD**	**pMCI due to AD**	**AD dementia**
Potentially eligible	42	38	150
Classified as not eligible	16	6	17
Percentage	38.1	15.8	11.3
Sensitivity	0.72	0.86	0.90

*pMCI* progressive MCI cases, patients who had progressive disease after 12-month follow up in the predictND cohort, *sMCI* stable MCI cases, patients who did not progress after 12-month follow up *AD* Alzheimer’s disease Dementia

### Clinical application

**Fig. 5:**
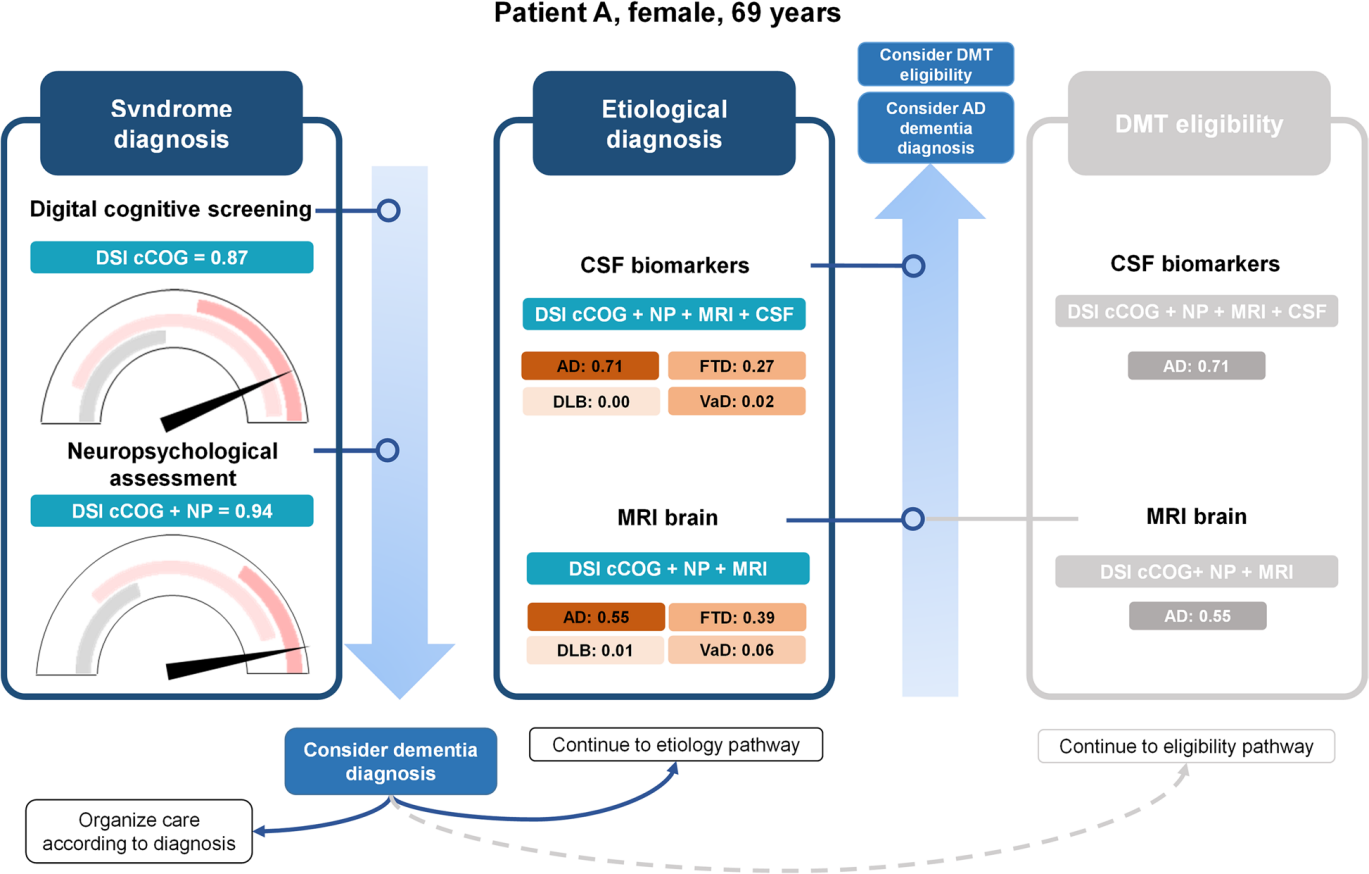
Example of the stepwise approach in clinical practice. Abbreviations: DSI: disease state index, cCOG: computerized cognitive screening, NP: neuropsychological and functional assessment, CN: cognitively normal, MCI: mild cognitive impairment, MMSE: mini-mental state examination, CSF: cerebrospinal fluid, MRI: magnetic resonance imaging, AD: Alzheimer’s disease, DLB: dementia with Lewy bodies, FTD: frontotemporal dementia, VaD: vascular dementia

To illustrate the practical application of the stepwise testing process using the CDSS, we present an example of the stepwise approach in clinical practice, using a fictive patient (Fig. 5). Patient A is a 69-year-old female who presented to the memory clinic with memory complaints. She underwent cCOG, and based on her cCOG results (DSI = 0.87), she was classified as MCI with an indeterminate DSI value, and it was recommended to continue to the next step to confirm the syndrome diagnosis. As such, cognitive and functional assessments were performed, after which the DSI increased to 0.94, indicating a greater likelihood of dementia. If the main focus is on syndrome diagnosis, the clinician may choose to conclude the diagnostic process. Alternatively, the clinician can continue to investigate the etiology, starting with the addition of MRI. The MRI results revealed the highest probability for AD as the underlying etiology (0.55). Since this value was below the predefined cutoff of 0.6, the clinician might decide to proceed with CSF testing. When CSF was subsequently is added, the probability of AD increased to 0.71, which concurrently reduced the likelihood of the other diseases. Note that this patient would also be selected for CSF testing to assess eligibility for DMT given that the DSI cutoff for AD after MRI exceeded the defined cutoff (> 0.1), and none of the other diagnoses had a DSI value of > 0.8.

## Discussion

In an era where disease-modifying therapies are becoming available for the first time, timely and accurate diagnosis of dementia is more important than ever. A data-driven tool can be beneficial for assisting clinicians in determining which tests add value in different clinical contexts. In this study, we applied a data-driven stepwise testing approach using a CDSS in three clinical scenarios to assess its potential to guide diagnostic decision-making. The stepwise testing approach results indicated that different scenarios require different quantities of diagnostic tests and thus reduce the number of tests required while maintaining diagnostic accuracy. Such an approach holds promise for ensuring that the diagnosis of dementia is accessible and affordable in the future. For the purpose of this proof-of-principle study, we divided the scenarios into three separate clinical scenarios. In real-world practice, the three could be combined into one harmonized workflow, as illustrated by the hypothetical patient case.

To address the challenges healthcare systems face with increasing patient volumes due to the growing prevalence of dementia and the introduction of DMTs and the significant national and regional differences in the accessibility and availability of diagnostic services it is crucial to improve diagnostic strategies [8, 47–49]. An ideal diagnostic pathway is tailored to the needs of both individual patients and clinicians, while being efficient at the same time. In this study, we assessed efficient pathways for three clinical scenarios based on different goals that clinicians have for which they deploy diagnostic testing. These different situations define an important role for a CDSS that can be flexible in the way it recommends ancillary testing to assist with the interpretation of complex, multimodal diagnostic data to provide accurate diagnostic predictions derived from reference populations. Although the diagnostic accuracies in this study were already decent, the CDSS could be further extended to increase the accuracy even further by incorporating other data, such as the extended cCOG version for DLB [50], and amyloid PET [13]. Currently, it is being extended by the development of FDG-PET and DaT-scan modules.

Timely diagnosis and early identification of patients eligible for DMT require biomarkers of amyloid pathology, but in regular clinical settings, only a small fraction of potentially eligible patients receive a biomarker-based confirmatory diagnosis [10]. Blood-based biomarkers (BBB) are rapidly evolving and have the potential to provide accessible evaluation of AD pathology [51, 52]. It is conceivable that for scenario 2 – differential diagnosis – BBBs may quickly replace CSF biomarkers [51]. In [53], it was suggested that CSF or amyloid PET confirmation is needed only for the intermediate probability group based on BBB results. The same concept of the high/ intermediate/low probability was followed for cognitive testing in this study and illustrated in Fig. 1. However, as BBBs are not yet widely in clinical use, it is still necessary to conduct CSF or amyloid-PET investigations when determining DMT eligibility. Therefore, it is highly relevant to find efficient ways to conduct CSF assessments for the right patient at the right time. We demonstrated that a stepwise approach can reduce the number of diagnostic tests while maintaining diagnostic accuracy. For example, our approach required only 61% of all MCI and dementia patients to undergo amyloid testing to identify potentially eligible patients, while still identifying 84% of the truly eligible patients. The 16% of patients who were missed were probably missed due to mixed pathologies. Nonetheless, one should note that our approach did not consider some of the factors that could render a patient ineligible, such as medication use or imaging features such as microbleeds [5, 6]. Improving the underlying models, also with respect to the other eligibility criteria, is currently ongoing in the European PROMINENT study [54].

The early and accurate identification of individuals who may develop dementia due to AD, i.e., ‘preclinical disease’ [55], and who are eligible for DMT requires the implementation of broader large-scale screening measures and cost-effective detection and diagnostic tools [6, 48]. In this regard, the development of digital cognitive test tools holds considerable promise [56]. In this study, we showed that (digital) cognitive screening is a powerful tool for identifying cognitively normal individuals who are not eligible for treatment. This can importantly support primary care in their role as a ‘gatekeeper’, ensuring that only those who need it are referred to memory clinics. However, primary care physicians often have limited time and resources available [9]. Therefore, using digital cognitive tests, such as cCOG, as a first step, at home can alleviate the burden on general practitioners [15]. Although cCOG has shown high accuracy in detecting cognitively healthy participants and dementia patients its ability to detect earliest subtle cognitive changes remains to be elucidated [15, 50].

Our data-driven approach is limited in that it does not consider the preferences and wishes of patients and care partners, or clinical impressions of clinicians. However, the results of our study indicate that using a stepwise approach has the potential to complement patient-centered biomarker-based workflows for specialist outpatient services as recently defined by a European task force [19], by offering data-driven and visually supported diagnostic probabilities for clinicians. Furthermore, patients and their care partners have their own needs and preferences regarding diagnostic testing, which are often not addressed during the consultation [12]. Stepwise diagnostic testing, along with the graphical counterpart, which makes interpretation of results to clinicians more transparent, and the option to visualize test results in easy-to-understand patient report [57], can guide clinicians in explaining diagnostic tests or test results, enhancing patient comprehension and contributing to shared decision-making.

Another often mentioned and important limitation of data-driven CDSSs is that they require multiple pieces of data that are not readily available to the clinician, or that the models are too complex, which limits their clinical footprint [16]. The CDSS should be intuitive (i.e., not a complex black box), time-efficient, and assist rather than replace the clinician [58]. The latter also means that if the tool’s result conflicts with the clinician’s opinion, the clinician would always have the final say and could choose to disregard the tool's outcome. Although the CDSS that we used in this study was developed with the aim of meeting these criteria and has been validated in clinical practice, it is not yet widely used in daily practice [17]. The implementation of new technologies in existing clinical workflows is often hindered by the need for integration with electronic patient files, a lack of experience with these tools and high workloads hindering adequate learning of how to use a tool. In other words, tools need to be tailored to real-world practices and meet the needs and preferences of clinicians. Follow-up research focusing on the implementation of the CDSS in daily practice should address these main requirements.

### Strengths and limitations

A major strength of our study is the use of data from two different memory clinic cohorts. All patients came to the memory clinic seeking medical help. The data thus reflect real-life clinical patients. In each clinic, patients were diagnosed using a comprehensive clinical workup, and we were able to use measures that overlapped. Since ground-truth diagnosis was obtained from a clinical diagnosis, not from a neuropathological confirmation, the true accuracy cannot be determined. However, the clinical workup was comprehensive; the material section describes only the data that were used in this study, but more comprehensive data were available for clinical diagnosis, and all clinical diagnoses were made in tertiary memory clinics through a consensus meeting in the ADC and after 12 months of follow-up in the PredictND cohort. In other words, the ground truth represents the current state of the art in clinical diagnosis. Another limitation is that we categorized patients as having a single pathology, while on average 20–40% of patients have multiple pathologies underlying their dementia diagnosis. The fact that we were able to correctly classify 80% of patients in the etiology scenario may be attributed to mixed pathology.

Finally, a limitation of this study is the choice of cutoffs applied in the stepwise approach. The cutoff values were selected ad hoc with the aim of, for example, emphasizing sensitivity over specificity in a certain scenario. Although other strategies could have been applied to optimize the use of cutoffs, we did not employ these in the current study for the purpose of readability and clinical applicability. Future research should address optimization strategies for optimal cutoff values. The stepwise approach shows promise in reducing the number of diagnostic tests performed. However, we did not consider any costs in the present study. To pursue the clinical application of a data-driven approach in real-world practice, a cost-efficiency study is warranted.

## Conclusion

The anticipated challenges in real-world dementia practices call for improved diagnostic strategies to establish a tailored diagnostic pathway that meets the specific needs of individual patients and clinicians while keeping dementia diagnosis accessible and affordable. In this study we demonstrated the promising value of a stepwise, data-driven approach for reducing the number of diagnostic tests required while maintaining diagnostic accuracy.

## Supplementary Information

Supplementary Material 1

## Data Availability

No datasets were generated or analysed during the current study.

## Abbreviations

AD: Alzheimer's disease
ADC: Amsterdam Dementia Cohort
APS: Anterior–posterior score
Aβ42: Amyloid β1-42
BBBs: Blood-based biomarkers
CDSS: Clinical decision support system
CERAD: Consortium to Establish a Registry for Alzheimer’s Disease word list memory test
cFazekas: Computed Fazekas
cGCA: Computed global cortical atrophy
cMTA: Computed medial temporal lobe atrophy
CN: Cognitively normal
CSF: Cerebrospinal fluid
DMT: Disease-modifying therapy
DLB: Dementia with Lewy bodies
DSI: Disease state index
ELISA: Enzyme-linked immunosorbent assays
FLAIR: Fluid-attenuated inversion recovery
FTD: Frontotemporal dementia
GDS: Geriatric Depression Scale
MCI: Mild cognitive impairment
MMSE: Mini-Mental State Examination
MRI: Magnetic resonance imaging
NP: Neuropsychological and functional assessment
NPI: Neuropsychiatric Inventory
PET: Positron emission tomography
p-tau: Phosphorylated tau
RAVLT: Rey auditory verbal learning task
ROI: Region of interest
SCD: Subjective cognitive decline
TMT-A/B: Trail Making Test A and B
t-tau: Total tau
VaD: Vascular dementia
WMH: White matter hyperintensities

## References

[1] 2016 Alzheimer's disease facts and figures. Alzheimers Dement, 2016. 12(4): p. 459-509. 10.1016/j.jalz.2016.03.001 27570871

[2] Robinson L, Tang E, Taylor JP. Dementia: timely diagnosis and early intervention. BMJ. 2015;350:h3029. 26079686 10.1136/bmj.h3029 PMC4468575

[3] van Maurik, I.S., et al., A more precise diagnosis by means of amyloid PET contributes to delayed institutionalization, lower mortality, and reduced care costs in a tertiary memory clinic setting. Alzheimer's & Dementia, 2022. n/a(n/a). 10.1002/alz.12846 36419238

[4] Ford E, Milne R, Curlewis K. Ethical issues when using digital biomarkers and artificial intelligence for the early detection of dementia. WIREs Data Min Knowl Discovery. 2023;13(3):e1492. 10.1002/widm.1492 PMC10909482 38439952

[5] Cummings J, et al. Aducanumab: appropriate use recommendations. J Prev Alzheimers Dis. 2021;8(4):398–410. 34585212 10.14283/jpad.2021.41 PMC8835345

[6] Cummings J, et al. Lecanemab: appropriate use recommendations. J Prev Alzheimers Dis. 2023;10(3):362–77. 37357276 10.14283/jpad.2023.30 PMC10313141

[7] Hazan J, Liu KY, Fox NC, Howard R. Online clinical tools to support the use of new plasma biomarker diagnostic technology in the assessment of Alzheimer’s disease: a narrative review. Brain Commun. 2023;5(6):fcad32. 10.1093/braincomms/fcad322 PMC10715781 38090277

[8] Hlavka JP, Mattke S, Liu JL. Assessing the preparedness of the health care system infrastructure in six European countries for an Alzheimer’s treatment. Rand Health Q. 2019;8(3):2. 31205802 10.7249/rhq-08-03-02 PMC6557037

[9] Hampel H, et al. Designing the next-generation clinical care pathway for Alzheimer’s disease. Nature Aging. 2022;2(8):692–703. 37118137 10.1038/s43587-022-00269-x PMC10148953

[10] Epelbaum S, et al. How many patients are eligible for disease-modifying treatment in Alzheimer’s disease? A French national observational study over 5 years. BMJ Open. 2019;9(6): e029663. 31239309 10.1136/bmjopen-2019-029663 PMC6597622

[11] Gruters AAA, et al. Development of memory clinics in the Netherlands over the last 20 years. Int J Geriatr Psychiatry. 2019;34(8):1267–74. 31034652 10.1002/gps.5132 PMC6767517

[12] Visser LNC, et al. Motivations of patients and their care partners for visiting a memory clinic. A qualitative study. Patient Educ Counseling. 2023;111:107693. 10.1016/j.pec.2023.107693 36913778

[13] Rhodius-Meester HFM, et al. Selection of memory clinic patients for CSF biomarker assessment can be restricted to a quarter of cases by using computerized decision support, without compromising diagnostic accuracy. PLoS ONE. 2020;15(1):e0226784. 31940390 10.1371/journal.pone.0226784 PMC6961870

[14] Galvin JE, et al. Early stages of Alzheimer’s disease: evolving the care team for optimal patient management. Front Neurol. 2020;11:592302. 33551954 10.3389/fneur.2020.592302 PMC7863984

[15] Rhodius-Meester HFM, et al. cCOG: a web-based cognitive test tool for detecting neurodegenerative disorders. Alzheimers Dement (Amst). 2020;12(1):e12083. 32864411 10.1002/dad2.12083 PMC7446945

[16] Sutton RT, et al. An overview of clinical decision support systems: benefits, risks, and strategies for success. npj Digital Med. 2020;3(1):17. 10.1038/s41746-020-0221-y PMC7005290 32047862

[17] Bruun M, et al. Impact of a clinical decision support tool on dementia diagnostics in memory clinics: the predictnd validation study. Curr Alzheimer Res. 2019;16(2):91–101. 30605060 10.2174/1567205016666190103152425

[18] Lam J, Mattke S. Memory care approaches to better leverage capacity of dementia specialists: a narrative synthesis. Neurodegener Dis Manag. 2021;11(3):239–50. 33966489 10.2217/nmt-2020-0038

[19] Frisoni GB, et al. European intersocietal recommendations for the biomarker-based diagnosis of neurocognitive disorders. Lancet Neurol. 2024;23(3):302–12. 38365381 10.1016/S1474-4422(23)00447-7

[20] Tolonen A, et al. Data-driven differential diagnosis of dementia using multiclass disease state index classifier. Front Aging Neurosci. 2018;10:111. 29922145 10.3389/fnagi.2018.00111 PMC5996907

[21] van der Flier WM, Scheltens P. Amsterdam dementia cohort: performing research to optimize care. J Alzheimers Dis. 2018;62(3):1091–111. 29562540 10.3233/JAD-170850 PMC5870023

[22] McKhann GM, et al. The diagnosis of dementia due to Alzheimer’s disease: recommendations from the national institute on aging-Alzheimer’s association workgroups on diagnostic guidelines for Alzheimer’s disease. Alzheimers Dement. 2011;7(3):263–9. 21514250 10.1016/j.jalz.2011.03.005 PMC3312024

[23] Jack CR Jr, et al. NIA-AA research framework: toward a biological definition of Alzheimer’s disease. Alzheimers Dement. 2018;14(4):535–62. 29653606 10.1016/j.jalz.2018.02.018 PMC5958625

[24] Rascovsky K, et al. Sensitivity of revised diagnostic criteria for the behavioural variant of frontotemporal dementia. Brain. 2011;134(Pt 9):2456–77. 21810890 10.1093/brain/awr179 PMC3170532

[25] Roman GC, et al. Vascular dementia: diagnostic criteria for research studies. Report of the NINDS-AIREN International Workshop. Neurology. 1993;43(2):250–60. 8094895 10.1212/wnl.43.2.250

[26] McKeith IG, et al. Diagnosis and management of dementia with Lewy bodies fourth consensus report of the DLB Consortium. Neurology. 2017;89(1):88–100. 28592453 10.1212/WNL.0000000000004058 PMC5496518

[27] Petersen RC. Mild cognitive impairment as a diagnostic entity. J Intern Med. 2004;256(3):183–94. 15324362 10.1111/j.1365-2796.2004.01388.x

[28] Jessen F, et al. A conceptual framework for research on subjective cognitive decline in preclinical Alzheimer’s disease. Alzheimers Dement. 2014;10(6):844–52. 24798886 10.1016/j.jalz.2014.01.001 PMC4317324

[29] Folstein MF, Folstein SE, McHugh PR. Mini-mental state". A practical method for grading the cognitive state of patients for the clinician. J Psychiatr Res. 1975;12(3):189–98. 1202204 10.1016/0022-3956(75)90026-6

[30] Schoenberg MR, et al. Test performance and classification statistics for the rey auditory verbal learning test in selected clinical samples. Arch Clin Neuropsychol. 2006;21(7):693–703. 16987634 10.1016/j.acn.2006.06.010

[31] Morris JC, et al. The Consortium to Establish a Registry for Alzheimer’s Disease (CERAD). Part I. Clinical and neuropsychological assessment of Alzheimer’s disease. Neurology. 1989;39(9):1159–65. 2771064 10.1212/wnl.39.9.1159

[32] Reitan RM. Validity of the trail making test as an indicator of organic brain damage. Percept Mot Skills. 1958;8(3):271–6.

[33] Van der Elst W, Van Boxtel MP, Van Breukelen GJ, Jolles J. Normative data for the animal, profession and Letter M Naming verbal fluency tests for Dutch speaking participants and the effects of age, education, and sex. J Int Neuropsychol Soc. 2006;12(1):80–9. 16433947 10.1017/S1355617706060115

[34] Wechsler D, WAIS-III: Administration and scoring manual: Wechsler adult intelligence scale. 1997: Psychological Corporation.

[35] Yesavage JA, et al. Development and validation of a geriatric depression screening scale: a preliminary report. J Psychiatr Res. 1982;17(1):37–49. 7183759 10.1016/0022-3956(82)90033-4

[36] Cummings JL, et al. The neuropsychiatric inventory. comprehensive assessment of psychopathology in dementia. Neurology. 1994;44(12):2308–2308. 7991117 10.1212/wnl.44.12.2308

[37] Feldman H, et al. The disability assessment for dementia scale: a 12-month study of functional ability in mild to moderate severity Alzheimer disease. Alzheimer Dis Assoc Disord. 2001;15(2):89–95. 11391090 10.1097/00002093-200104000-00008

[38] Koikkalainen J, et al. Differential diagnosis of neurodegenerative diseases using structural MRI data. Neuroimage Clin. 2016;11:435–49. 27104138 10.1016/j.nicl.2016.02.019 PMC4827727

[39] Bruun M, et al. Detecting frontotemporal dementia syndromes using MRI biomarkers. Neuroimage Clin. 2019;22:101711. 30743135 10.1016/j.nicl.2019.101711 PMC6369219

[40] Koikkalainen JR, et al. Automatically computed rating scales from MRI for patients with cognitive disorders. Eur Radiol. 2019;29(9):4937–47. 30796570 10.1007/s00330-019-06067-1

[41] Willemse EAJ, et al. Diagnostic performance of Elecsys immunoassays for cerebrospinal fluid Alzheimer’s disease biomarkers in a nonacademic, multicenter memory clinic cohort: the ABIDE project. Alzheimers Dement (Amst). 2018;10:563–72. 30406175 10.1016/j.dadm.2018.08.006 PMC6215060

[42] Tijms BM, et al. Unbiased approach to counteract upward drift in cerebrospinal fluid amyloid-β 1–42 analysis results. Clin Chem. 2018;64(3):576–85. 29208658 10.1373/clinchem.2017.281055

[43] Mulder C, et al. Amyloid-β(1–42), total Tau, and phosphorylated tau as cerebrospinal fluid biomarkers for the diagnosis of Alzheimer disease. Clin Chem. 2010;56(2):248–53. 19833838 10.1373/clinchem.2009.130518

[44] Duits FH, et al. The cerebrospinal fluid “Alzheimer profile”: easily said, but what does it mean? Alzheimers Dement. 2014;10(6):713-723 e2. 24721526 10.1016/j.jalz.2013.12.023

[45] Mattila J, et al. A disease state fingerprint for evaluation of Alzheimer’s disease. J Alzheimers Dis. 2011;27(1):163–76. 21799247 10.3233/JAD-2011-110365

[46] Hofmann H, Wickham H, Kafadar K. Letter-value plots: boxplots for large data. J Comput Graph Stat. 2017;26(3):469–77.

[47] Jönsson L, et al. The affordability of lecanemab, an amyloid-targeting therapy for Alzheimer’s disease: an EADC-EC viewpoint. The Lancet Regional Health - Europe. 2023;29:100657. 37251789 10.1016/j.lanepe.2023.100657 PMC10220264

[48] Wahlberg K., et al., People get ready! A new generation of Alzheimer's therapies may require new ways to deliver and pay for healthcare. J Intern Med, 2023. n/a(n/a). 10.1111/joim.13759 38098165

[49] Alzheimer Europe. Alzheimer Europe call for action on anti-amyloid therapies for Alzheimer’s disease. 2023. Available from: https://www.alzheimer-europe.org/sites/default/files/2024-01/2023-09_ae_call_for_action_on_anti-amyloid_treatments.pdf. Cited 2024 23/01. 10.14283/jpad.2024.37 38374732

[50] van Gils AM, et al. Optimizing cCOG, a Web-based tool, to detect dementia with Lewy Bodies. Alzheimers Dement (Amst). 2022;14(1):e12379. 36569383 10.1002/dad2.12379 PMC9773307

[51] Ashton NJ, et al. Diagnostic Accuracy of a Plasma Phosphorylated Tau 217 Immunoassay for Alzheimer Disease Pathology. JAMA Neurology, 2024. 10.1001/jamaneurol.2023.5319 PMC10804282 38252443

[52] Barthélemy NR. et al. Highly Accurate Blood Test for Alzheimer’s Disease Comparable or Superior to Clinical CSF Tests. Nature Medicine. 2024. 10.1038/s41591-024-02869-z PMC11031399 38382645

[53] Hansson O, Blennow K, Zetterberg H, Dage J. Blood biomarkers for Alzheimer’s disease in clinical practice and trials. Nat Aging. 2023;3(5):506–19. 37202517 10.1038/s43587-023-00403-3 PMC10979350

[54] Liss JL, et al. Practical recommendations for timely, accurate diagnosis of symptomatic Alzheimer’s disease (MCI and dementia) in primary care: a review and synthesis. J Intern Med. 2021;290(2):310–34. 33458891 10.1111/joim.13244 PMC8359937

[55] Vos SJ, et al. Preclinical Alzheimer’s disease and its outcome: a longitudinal cohort study. Lancet Neurol. 2013;12(10):957–65. 24012374 10.1016/S1474-4422(13)70194-7 PMC3904678

[56] Öhman F, et al. Current advances in digital cognitive assessment for preclinical Alzheimer’s disease. Alzheimers Dement (Amst). 2021;13(1):e12217. 34295959 10.1002/dad2.12217 PMC8290833

[57] van Gils AM, et al. Development and design of a diagnostic report to support communication in dementia: Co-creation with patients and care partners. Alzheimer’s Dementia. 2022;14(1):e12333. 36092691 10.1002/dad2.12333 PMC9446898

[58] van Gils AM, et al. Assessing the views of professionals, patients, and care partners concerning the use of computer tools in memory clinics: international survey study. JMIR Form Res. 2021;5(12):e31053. 34870612 10.2196/31053 PMC8686488

