# Supplementary material for: Computerized decision support to optimally funnel patients through the diagnostic pathway for dementia
Source: Alzheimers Res Ther. 2024 Nov 26;16:256. doi: 10.1186/s13195-024-01614-5 (PMC11590510; doi:10.1186/s13195-024-01614-5)
Supplement: Supplementary file 1 — Supplementary Material 1 [file 13195_2024_1614_MOESM1_ESM.docx]

**Supplementary files**

**Appendix A: Disease State Index Classifier**

The DSI is composed of two components: fitness and relevance. Fitness of test i, as a function of the test result value x, is defined as

$$f_{i}\left( x \right)= \frac{{FNR}_{i}(x)}{{FNR}_{i}\left( x \right)+{FPR}_{i}\left( x \right)}$$

where FNR and FPR stand for the rate of false negatives and false positives, respectively, in the reference data with a classification cut-off value *x*. The fitness value zero (one) indicates a high similarity to the negative (positive) group and the value 0.5 means that the test result is inconclusive. *Relevance* is calculated for a test *i* as

$$\text{relevance}_{i}=\text{sensitivity}_{i}+\text{specificity}_{i}-1.$$

Relevance is a value between 0 and 1 telling how well test *i* separates the groups in the training set. The DSI value for multiple test results is the average of fitness values weighted with normalized relevance values:

$$\text{DSI}= \frac{\sum\text{relevance}_{i}\times{fitness}_{i}}{\sum\text{relevance}_{i}}$$

**Supplementary Figure 1. Scenario 1 – Sensitivity and specificity curves according to DSI cutoff values using the two-class classifier (CN/dementia) for each step in Scenarios 1A and 1B.**


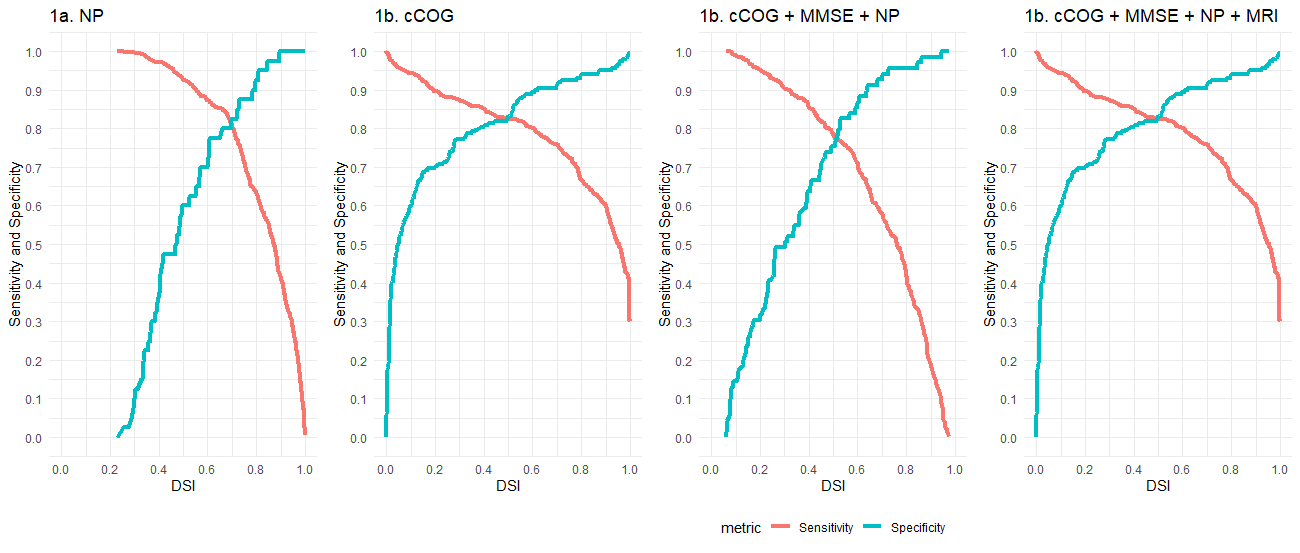


**Supplementary Figure 2. Scenario 2, step 1. cCOG and NP – Sensitivity and specificity curves according to DSI cutoff values using the two-class classifier (CN/dementia).**


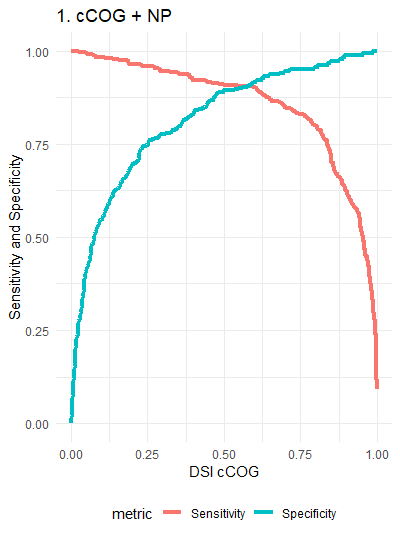


**Supplementary Figure 3. Scenario 2, step 2. cCOG, NP, and MRI – Sensitivity and specificity curves according to DSI cutoff values using two-class classifiers for each diagnosis (AD, FTD, VaD, DLB), in which the diagnosis is the positive class.**


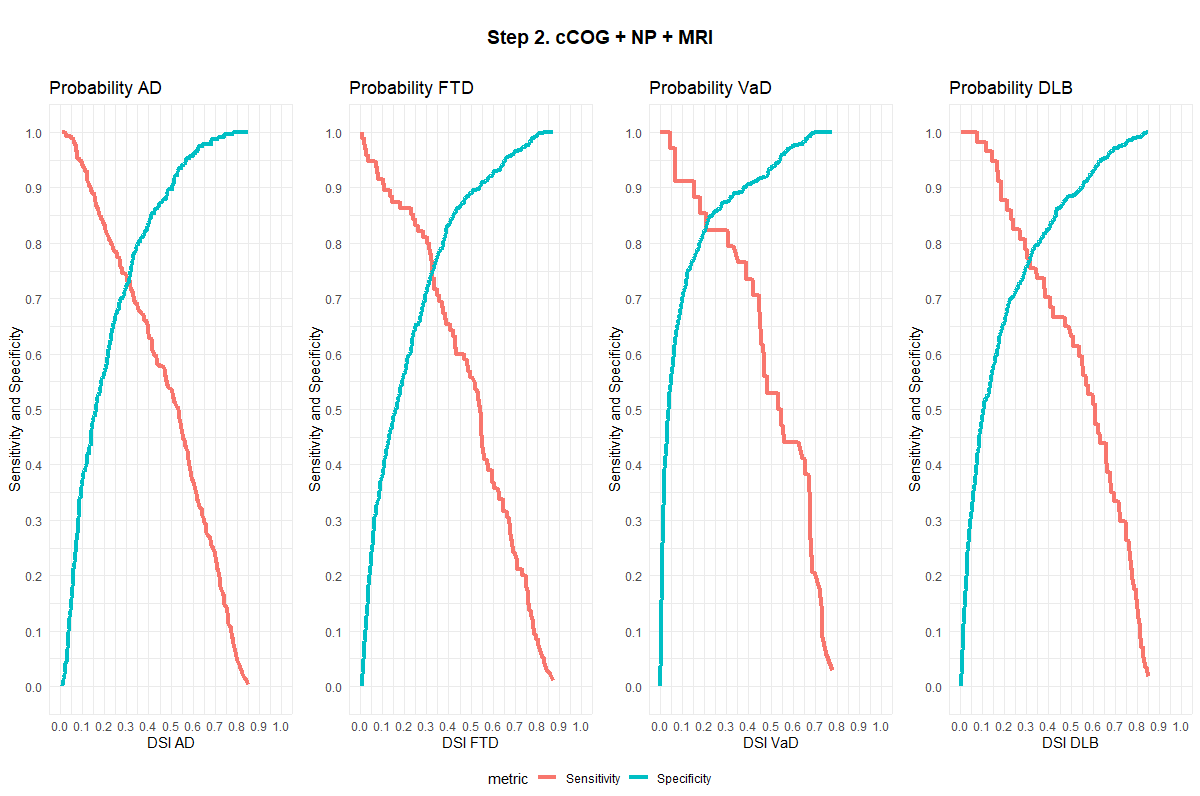


**Supplementary Figure 3. Scenario 2, step 2. cCOG, NP, MRI and CSF – Sensitivity and specificity curves according to DSI cutoff values using two-class classifiers for each diagnosis (AD, FTD, VaD, DLB), in which the diagnosis is the positive class.**


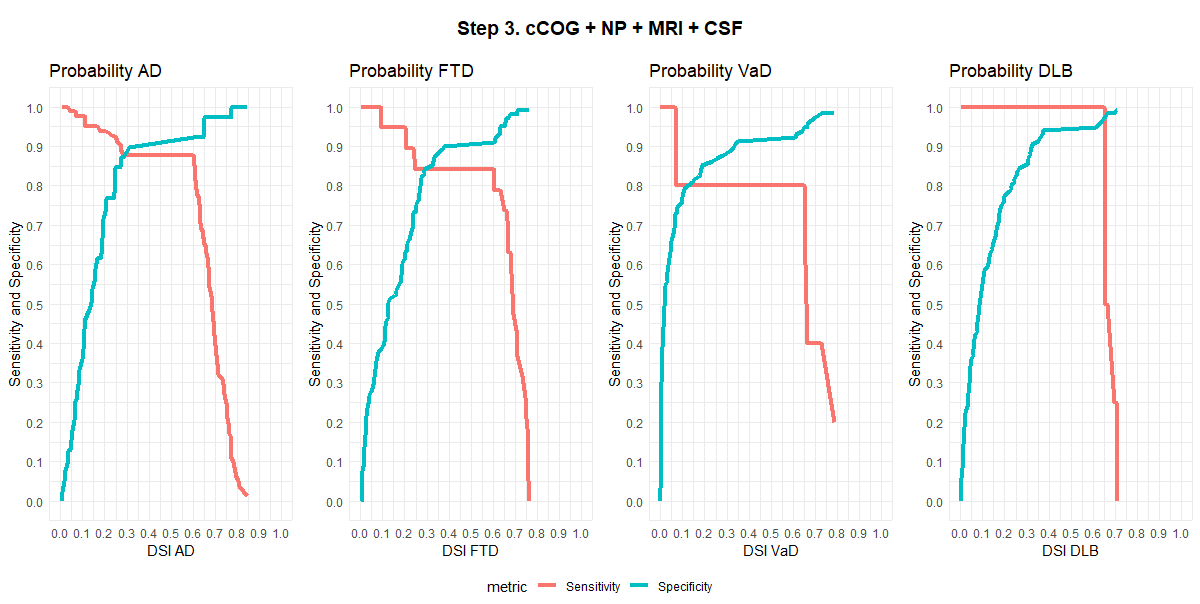


**Supplementary Figure 5. Scenario 3 – Sensitivity and specificity curves according to DSI cutoff values using a two-class classifier (CN/dementia or AD/other), for each subsequent step in the eligibility scenario.** Note: the reference diagnosis is MCI/dementia due to AD, MMSE ≥22, cFazekas <2.5, and positive amyloid biomarker.

**
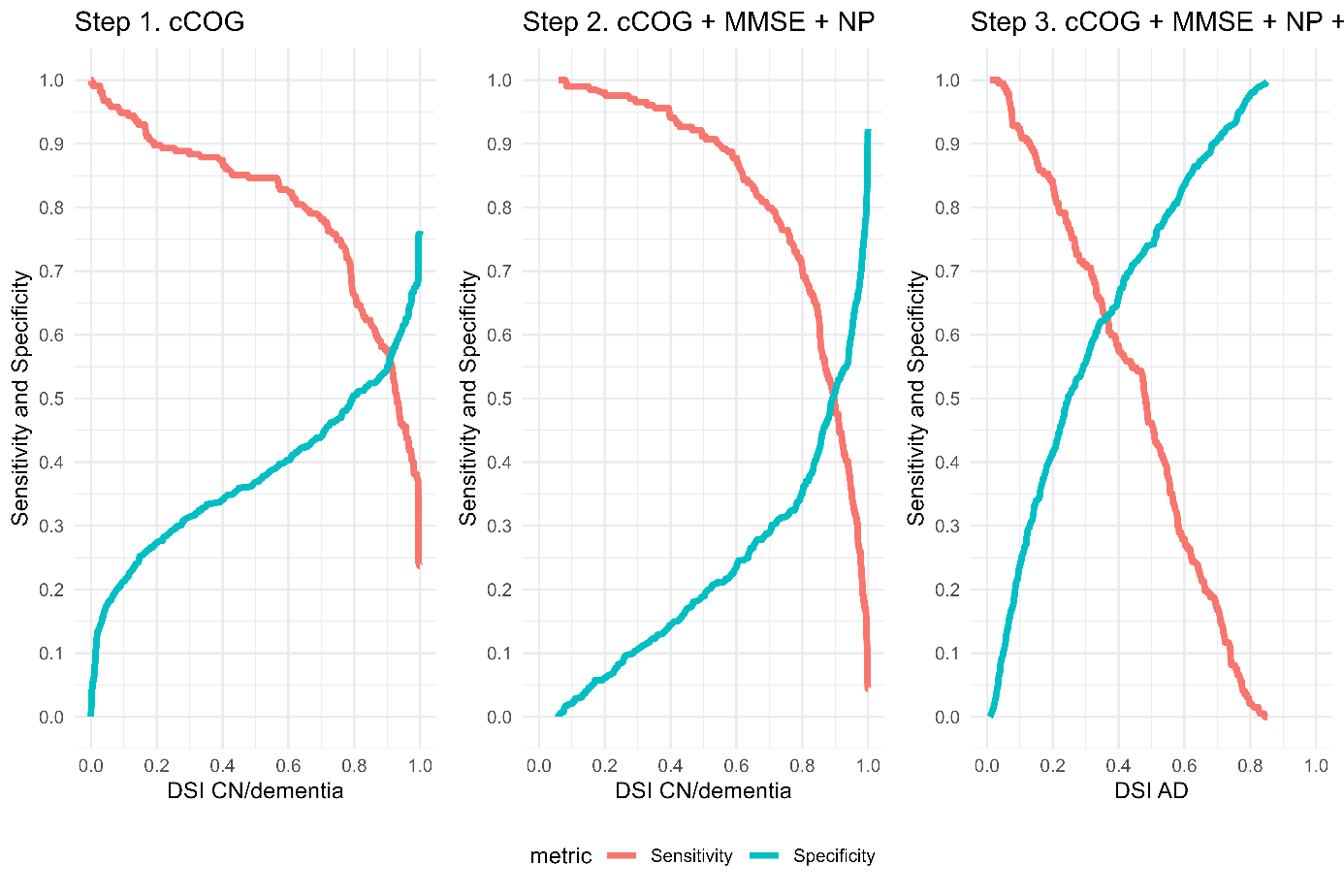
**

**Supplementary Figure 6. Scenario 2 - Letter value plots for distribution of DSI values among diagnostic groups for steps 2 and 3.**

**
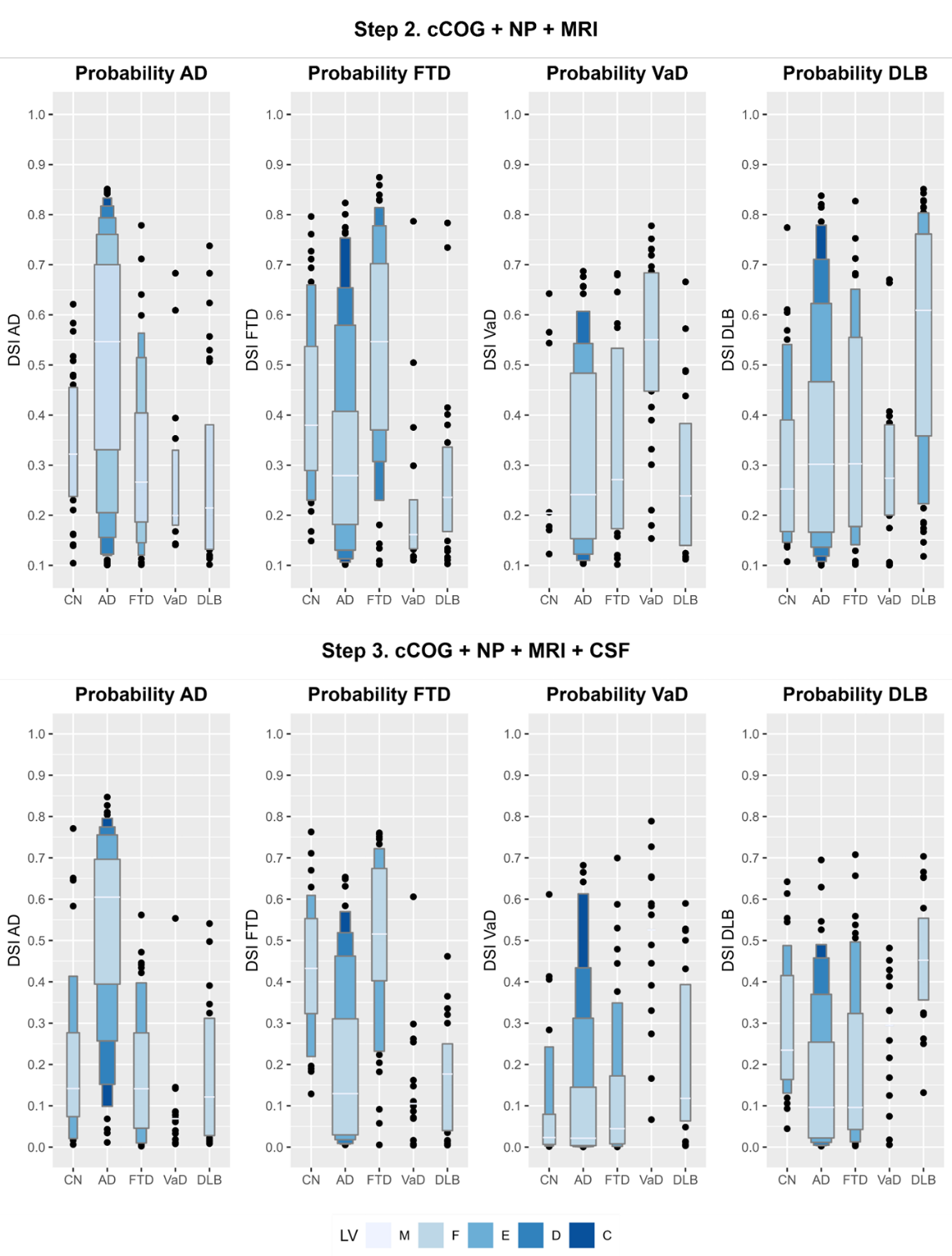
**

NOTE: The figures show letter-value plots for DSI scores for each diagnosis. The x-axis represent the true diagnosis. The length of the first box (F) represents the interquartile range (IQR), which contains the middle 50% of the data in the group, the second box (E) 12,5% at each end, the third box covers (D) 6,25% on each end, and the fourth box (C) 3,13%. The longer the box, the greater the variability. *Abbreviations:* AD: Alzheimer’s disease, FTD: frontotemporal dementia, VaD: vascular dementia, DLB: dementia with Lewy bodies. cCOG: computerized cognitive screening, MRI: magnetic resonance imaging, NP: neuropsychological assessment, CSF: cerebrospinal fluid.

| **Supplementary Table 1. Confusion matrices for syndrome diagnosis for participants for whom real cCOG data was available** | | | | | | |  |
| --- | --- | --- | --- | --- | --- | --- | --- |
|  | **Scenario 1B. cCOG** | | | **Scenario 1B. cCOG+NP** | | | |
|  | **CN** | **MCI** | **DEM** | **CN** | **MCI** | **DEM** | |
| **CN** | 30 | 4 | 4 | 40 | 9 | 6 | |
| **MCI** | 15 | 20 | 17 | 4 | 11 | 8 | |
| **DEM** | 1 | 1 | 19 | 2 | 5 | 26 | |
| *Cut-offs* | <0.1 | 0.1-0.95 | >0.95 | <0.3 | 0.3-0.7 | >0.7 | |
| *Sensitivity* | 0.88 | 0.80 | 0.48 | 0.90 | 0.30 | 0.85 | |
| *Specificity* | 0.65 | 0.63 | 0.97 | 0.79 | 0.89 | 0.72 | |
| *Accuracy* |  | 0.62 |  |  | 0.72 |  | |
| In the confusion matrix each column represents the actual diagnosis and each row the diagnosis suggested by the classifier; the cells show the number of patients in each category. Abbreviations: CN: cognitively normal, MCI: mild cognitive impairment, cCOG: computerized cognitive testing, NP: neuropsychological and functional assessment, MRI: magnetic resonance imaging. | | | | | | |  |

**Supplementary Table 2. Scenario 2, step 1: confusion matrix of two-class classification (‘CN’ vs. ‘dementia’) using the computerized cognitive test, cCOG, cognitive and functional assessment**

|  | *Reference diagnosis* | | | | |
| --- | --- | --- | --- | --- | --- |
|  | **CN** | **AD** | **FTD** | **VaD** | **DLB** |
| **CN** | 141 | 4 | 12 | 1 | 3 |
| **Dementia** | 47 | 298 | 95 | 34 | 57 |

Note: the DSI-cutoff used for this classification was 0.25 for CN. *Abbreviations*: CN: cognitively normal, AD: Alzheimer’s disease, FTD: frontotemporal dementia, VaD: vascular dementia, DLB: dementia with Lewy bodies.

| **Supplementary Table 3. Scenario 2, step 2: confusion matrix for patients (n=275) receiving diagnosis after cCOG, cognitive testing and MRI.** | | | | | |
| --- | --- | --- | --- | --- | --- |
|  | *Reference diagnosis* | | | | |
|  | **CN** | **AD** | **FTD** | **VaD** | **DLB** |
| **AD** | 1 | 111 | 3 | 2 | 3 |
| **FTD** | 9 | 20 | 35 | 1 | 2 |
| **VaD** | 1 | 7 | 3 | 15 | 1 |
| **DLB** | 3 | 21 | 6 | 2 | 29 |
| *Sensitivity* | 0.91 | 0.68 | 0.59 | 0.71 | 0.76 |
| *Specificity* | 0.93 | 0.96 | 0.90 | 0.96 | 0.90 |

Note: the DSI-cutoff used for this classification was 0.6; a diagnosis of CN was not given in this step. *Abbreviations*: CN: cognitively normal, AD: Alzheimer’s disease, FTD: Frontotemporal dementia, VaD: vascular dementia, DLB: dementia with Lewy bodies.

| **Supplementary Table 4. Scenario 2, step 3. Confusion matrix for patients (n=120) receiving diagnosis after cCOG, cognitive testing, MRI and CSF** | | | | | |
| --- | --- | --- | --- | --- | --- |
|  | *Reference diagnosis* | | | | |
|  | **CN** | **AD** | **FTD** | **VaD** | **DLB** |
| **AD** | 3 | 71 | 0 | 0 | 0 |
| **FTD** | 5 | 3 | 16 | 1 | 0 |
| **VaD** | 1 | 5 | 1 | 4 | 0 |
| **DLB** | 2 | 2 | 2 | 0 | 4 |
| *Sensitivity* | 0.93 | 0.84 | 0.50 | 0.67 | 0.57 |
| *Specificity* | 0.84 | 0.98 | 0.96 | 0.97 | 0.97 |

Note: the DSI-cutoff used for this classification was 0.6; a diagnosis of CN was not given in this step. *Abbreviations*: CN: cognitively normal, AD: Alzheimer’s disease, FTD: Frontotemporal dementia, VaD: vascular dementia, DLB: dementia with Lewy bodies.

**Supplementary Table 5. Scenario 3 Confusion matrices of two-class classification to detect potential eligible patients for DMT, for A) all participants and B) participants for whom real cCOG data were available**

| **A. All** | | | **B. Real cCOG data** | | |
| --- | --- | --- | --- | --- | --- |
| **4A. cCOG** | *Reference diagnosis* | |  | *Reference diagnosis* | |
| *Cutoff 0.1* | **Not eligible** | **Potentially eligible** |  | **Not eligible** | **Potentially eligible** |
| **Not eligible** | 139 | 13 | **Not eligible** | 35 | 3 |
| **Potentially eligible** | 508 | 217 | **Potentially eligible** | 43 | 28 |
| *Sensitivity* | 0.94 | | *Sensitivity* | 0.95 | |
| *Specificity* | 0.21 | | *Specificity* | 0.45 | |
| **4B. cCOG + NP** | *Reference diagnosis* | |  | *Reference diagnosis* | |
| *Cutoff 0.3* | **Not eligible** | **Potentially eligible** |  | **Not eligible** | **Potentially eligible** |
| **Not eligible** | 54 | 9 | **Not eligible** | 14 | 3 |
| **Potentially eligible** | 454 | 208 | **Potentially eligible** | 29 | 25 |
| *Sensitivity* | 0.96 | | *Sensitivity* | 0.89 | |
| *Specificity* | 0.11 | | *Specificity* | 0.33 | |
| **4C. cCOG + NP + MRI** | *Reference diagnosis* | |  | *Reference diagnosis* | |
| *Cutoff 0.1* | **Not eligible** | **Potentially eligible** |  | **Not eligible** | **Potentially eligible** |
| **Not eligible** | 117 | 17 | **Not eligible** | 6 | 2 |
| **Potentially eligible** | 337 | 191 | **Potentially eligible** | 23 | 23 |
| *Sensitivity* | 0.92 | | *Sensitivity* | 0.92 | |
| *Specificity* | 0.26 | | *Specificity* | 0.21 | |
